# Tumor-secreted LCN2 impairs gastric cancer progression via autocrine inhibition of the 24p3R/JNK/c-Jun/SPARC axis

**DOI:** 10.1038/s41419-024-07153-z

**Published:** 2024-10-18

**Authors:** Zhixin Huang, Ying Li, Yan Qian, Ertao Zhai, Zeyu Zhao, Tianhao Zhang, Yinan Liu, Linying Ye, Ran Wei, Risheng Zhao, Zikang Li, Zhi Liang, Shirong Cai, Jianhui Chen

**Affiliations:** 1 Division of Gastrointestinal Surgery Center, the First Affiliated Hospital of Sun Yat-sen University, Guangzhou, 510080 Guangdong China; 2 Gastric Cancer Center, Sun Yat-sen University, Guangzhou, 510080 Guangdong China; 3 Laboratory of Surgery, the First Affiliated Hospital of Sun Yat-sen University, Guangzhou, 510080 Guangdong China; 4 Guangdong Provincial Key Laboratory of Microbial Safety and Health, National Health Commission Science and Technology Innovation Platform for Nutrition and Safety of Microbial Food, State Key Laboratory of Applied Microbiology Southern China, Institute of Microbiology, Guangdong Academy of Sciences, Guangzhou, 510070 China; 5 Department of General Surgery, Guangxi Hospital Division of The First Affiliated Hospital, Sun Yat-sen University, Nanning, 530000 Guangxi China

**Keywords:** Tumour biomarkers, Gastrointestinal cancer, Tumour-suppressor proteins

## Abstract

Gastric cancer (GC) is one of the most lethal malignancies worldwide. Despite extensive efforts to develop novel therapeutic targets, effective drugs for GC remain limited. Recent studies have indicated that Lipocalin (LCN)2 abnormalities significantly impact GC progression; however, its regulatory network remains unclear. Our study investigates the functional role and regulatory mechanism of action of LCN2 in GC progression. We observed a positive correlation between LCN2 expression, lower GC grade, and better prognosis in patients with GC. LCN2 overexpression suppressed GC proliferation and metastasis both in vitro and in vivo. Transcriptome sequencing identified secreted protein acidic and rich in cysteine (SPARC) as a pivotal downstream target of LCN2. Mechanistically, c-Jun acted as a transcription factor inducing SPARC expression, and LCN2 downregulated SPARC by inhibiting the JNK/c-Jun pathway. Moreover, LCN2 bound to its receptor, 24p3R, via autocrine signaling, which directly inhibited JNK phosphorylation and then inhibited the JNK/c-Jun pathway. Finally, analysis of clinical data demonstrated that SPARC expression correlated negatively with lower GC grade and better prognosis, and that LCN2 expression correlated negatively with p-JNK, c-Jun, and SPARC expression in GC. These findings suggest that the LCN2/24p3R/JNK/c-Jun/SPARC axis is crucial in the malignant progression of GC, offering novel prognostic markers and therapeutic targets.

## Introduction

Gastric cancer (GC) is the fifth most prevalent cancer worldwide and the third leading cause of cancer-related death [1, 2]. Although the prognosis of GC has improved with the development of comprehensive treatment, the 5-year survival rate for patients with GC remains below 40% [3]. In addition, the prognosis of metastatic, recurrent, and advanced GC remains unsatisfactory, as the mechanisms underlying GC occurrence and progression are unclear [4]. Therefore, uncovering of the etiology of GC, identification of novel diagnostic markers, and discovery of promising therapeutic targets are urgently required.

Lipocalin (LCN)2, also known as neutrophil gelatinase-associated lipocalin (NGAL), siderocalin, and 24p3, is a secreted protein initially identified as a crucial component of the innate immune system against microorganisms [5, 6]. LCN2/NGAL (LCN2 for short) is implicated in a variety of physiological roles, including hydrophobic ligand transport across cell membranes, immune response modulation, iron homeostasis maintenance, and epithelial cell differentiation promotion [5–7]. Meanwhile, LCN2 dysregulation has also been linked to a number of human diseases, such as obesity, metabolic syndrome, and cardiovascular disease [6, 8, 9]. Notably, recent studies have shown that LCN2 is abnormally expressed in a variety of cancers, including breast [10], colon [11, 12], pancreas cancers [13]. Furthermore, the oncological role of LCN2 in various cancers has been investigated, revealing its close association with cancer occurrence and development [11, 13, 14]. However, the role of action of LCN2 in GC remains unclear and controversial. Nishimura et al. reported that LCN2 inhibits EMT process via MMP2 downregulation, resulting in GC progression inhibition [15]. However, Xu et al. and Koh et al. suggested that LCN2 promotes GC progression via different molecular mechanisms [16, 17]. Notably, our previous single-cell RNA sequencing (scRNA-seq) data identified lower LCN2 expression levels in metastatic lymph nodes (LN) than in that in their paired primary GC sites, suggesting a potential relationship between LCN2 and GC progression [18]. Therefore, investigating the role of LCN2 in GC is paramount and warrants further study.

Secreted protein acidic and rich in cysteine (SPARC) is a unique matricellular glycoprotein associated with many biologic processes, including development, wound repair, tissue remodeling, angiogenesis, matrix cell adhesion, cell differentiation, proliferation, and migration [19, 20]. The role of SPARC in cancer has generated considerable interest owing to the fact that it functions not only its ability to modulate cell-cell and cell–matrix interactions, but its de-adhesive and growth inhibitory properties in non-transformed cells. The divergent actions of SPARC also led to questions regarding its complexity and how it regulates tumor growth [21–24]. For example, increased SPARC expression is strongly associated with tumor progression in gliomas and melanomas [25, 26], while some studies have reported SPARC as a tumor suppressor in neuroblastomas as well as ovarian and colorectal cancers [27–29]. Currently, the function of SPARC in GC progression is also unclear and conflicting. Most studies have shown that SPARC expression is upregulated in GC tissues, and upregulation of SPARC expression is negatively correlated with prognosis and patient survival [30, 31]. However, one study showed that SPARC is negatively correlated with clinical factors of GC and suppressed GC cell metastasis by decreasing MMP-7, MMP-9, N-cadherin, Sp1, and p-ERK1/2 expression [32]. Taken together, current studies do not provide a unifying picture of the function of SPARC in GC, nor explain the precise mechanism by which its expression is upregulated. Therefore, exploring the detailed roles and regulatory mechanisms of SPARC in GC is urgently required.

In this study, we investigated the functional role and regulatory mechanism of action of LCN2 in GC progression, and its cross-talk with underlying SPARC upregulation in GC. We demonstrated that LCN2 was aberrantly expressed in GC and correlated with a positive prognosis in patients with GC. LCN2 deletion promoted GC proliferation and metastasis both in vitro and in vivo. Mechanistically, LCN2/24p3R signaling inhibited the expression of SPARC by directly inactivating the JNK/c-Jun pathway. Our study reveals the functional role and regulatory mechanism of action of LCN2 in GC progression, suggesting its potential as a promising therapeutic target for GC treatment.

## Results

### LCN2 is aberrantly expressed in GC and correlates with the positive prognosis of patients

**Fig. 1:**
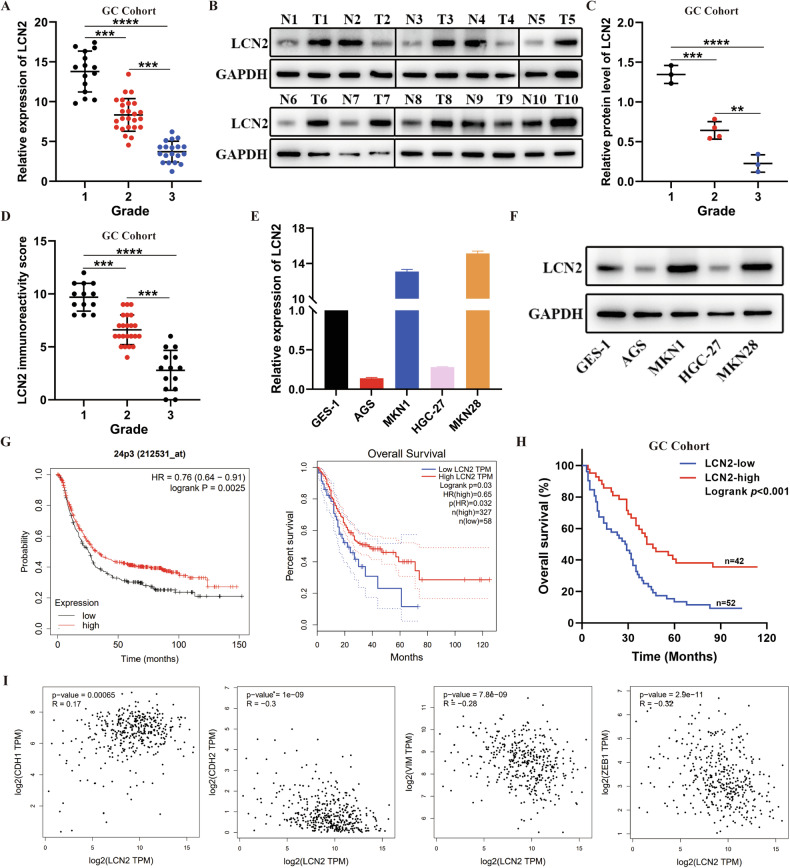
LCN2 is aberrantly expressed in GC and correlates with the positive prognosis of patients. **A** LCN2 mRNA expression was negatively correlated with GC grade in our cohort. **B** Detection of LCN2 protein levels in GC tissues and their paired adjacent normal tissues using western blot (*n* = 10). **C**, **D** LCN2 protein expression was negatively correlated with GC grade in our cohort. **E, F** mRNA and protein expression levels of LCN2 in GES-1 and various GC cells. **G** TCGA and KM-plot database showed that LCN2 overexpression correlated with better prognosis. **H** Kaplan–Meier survival analysis revealed that low expression of LCN2 is correlated with shorter overall survival times in our cohort (*n* = 94, *p* < 0.001; log-rank test). **I** Correlation analysis between LCN2 expression and CDH1, CDH2, VIM, and ZEB1 expression in GC from the TCGA database. Data are presented as mean ± SD of three independent experiments. *p < 0.05, **p < 0.01, ***p < 0.001.

To investigate the function and mechanism of action of LCN2 in GC progression, LCN2 expression levels were analyzed in GC and the adjacent normal tissues using the Cancer Genome Atlas (TCGA) database. The results indicated upregulation of *LCN2* expression in GC tissues (Supplementary Fig. 1A), with a negative correlation between LCN2 expression and GC grade (Supplementary Fig. 1B). To validate these findings, *LCN2* mRNA levels in a cohort of 59 paired GC tissue samples and their adjacent non-tumor tissues was examined. Consistent with the results of TGCA analysis, *LCN2* mRNA expression was upregulated in GC tissues compared to that in non-tumor tissues (*p* < 0.001, Supplementary Fig. 1C), and a significant negative correlation was observed between *LCN2* mRNA expression and GC grade (Fig. 1A). These findings were further confirmed at the protein level using western blotting and immunohistochemistry (IHC) (Fig. 1B–D and Supplementary Fig. 1D). In addition, GC cells with high proliferation and metastatic capabilities (AGS, HGC-27) exhibited lower LCN2 levels than GC cells with lower proliferation and metastatic capabilities (MKN1 and MKN28) (Fig. 1E, F). Altogether, these findings suggested aberrant LCN2 expression in GC and a potential negative association with GC progression.

Data from the TCGA database and Kaplan–Meier plotter were analyzed to understand the clinicopathological characteristics and prognostic value of LCN2 in GC. Survival analysis indicated that high LCN2 expression was associated with positive prognosis (Fig. 1G). Furthermore, 94 patients with GC in the tissue microarray were categorized into low (score 0–6) or high (score 7–12) LCN2 expression groups according to their IHC scores (Supplementary Fig. 1E). Consistent with the information in the database, patients with high LCN2 levels in primary tumors displayed significantly better prognoses (*p* < 0.001) (Fig. 1H). Moreover, high LCN2 expression correlated with smaller tumor sizes (*p* = 0.0038), fewer distant metastases (*p* = 0.0139), and LN metastases (*p* = 0.002) (Supplementary Table 1). A significant relationship between LCN2 expression and epithelial mesenchymal transition-related markers was also observed (Fig. 1I). Collectively, these results suggest that LCN2 loss may contribute to GC progression, and that its expression may have prognostic value for GC patients.

### LCN2 inhibits proliferation, migration, and invasion capabilities of GC cells in vitro

**Fig. 2:**
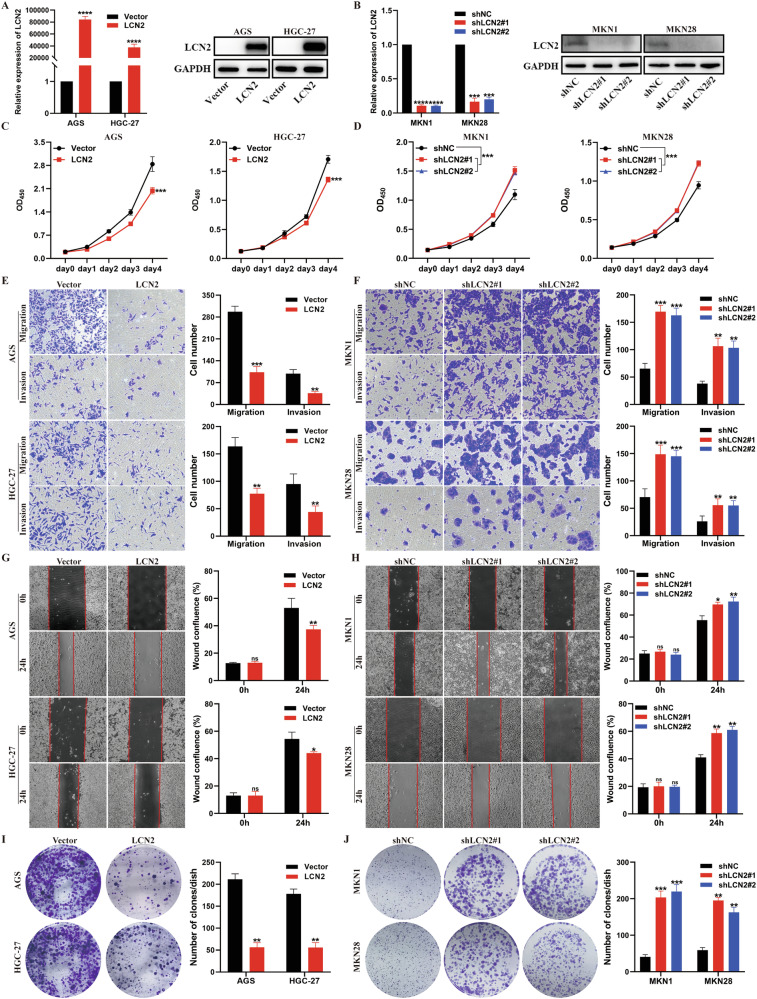
LCN2 inhibits proliferation, migration, and invasion capabilities of GC cells in vitro. **A** Measurement of LCN2 overexpression efficiencies in AGS and HGC-27 cells using RT-qPCR and western blot. **B** Measurement of LCN2 knockdown efficiencies in MKN1 and MKN28 cells using RT-qPCR and western blot. **C**, **D** LCN2 overexpression suppressed the proliferation of GC cells but LCN2 knockdown promotes as assessed by CCK-8 assay. **E**, **F** Representative images (left panel) and quantitative results (right panel) of transwell assay in transfected GC cells. **G**, **H** Representative images (left panel) and quantitative results (right panel) of wound healing assay in transfected GC cells. **I**, **J** Representative images (left panel) and quantitative results (right panel) colony formation assay in transfected GC cells. Data are presented as mean ± SD of three independent experiments. *p < 0.05, **p < 0.01, ***p < 0.001.

GC cell lines were selected for gain- or loss-of-function studies based on their low or high endogenous LCN2 levels. The efficiency of stable overexpression and knockdown of LCN2 was verified using reverse transcription-quantitative polymerase chain reaction (RT-qPCR) and western blotting (Fig. 2A, B and Supplementary Fig. 2A). The cell counting kit-8 (CCK-8) assay revealed that LCN2 overexpression inhibited GC cell proliferation (Fig. 2C and Supplementary Fig. 2B), while LCN2 knockdown showed the opposite effect (Fig. 2D and Supplementary Fig. 2C). Transwell and wound healing assays were performed to assess the effects of LCN2 on GC cell migration and invasion. The results demonstrated that LCN2 overexpression delayed wound closure and decreased migration and invasion abilities (Fig. 2E, G and Supplementary Fig. 2D, F). Conversely, these abilities were markedly enhanced after LCN2 knockdown (Fig. 2F, H and Supplementary Fig. 2E, G). Finally, colony formation assays revealed a reduction in colony number with LCN2 overexpression (Fig. 2I and Supplementary Fig. 2H), while LCN2 knockdown promoted colony formation (Fig. 2J and Supplementary Fig. 2I). Taken together, these results demonstrate that LCN2 inhibits GC cells proliferation, migration, and invasion capabilities in vitro.

### LCN2 suppresses GC growth and metastasis in vivo

**Fig. 3:**
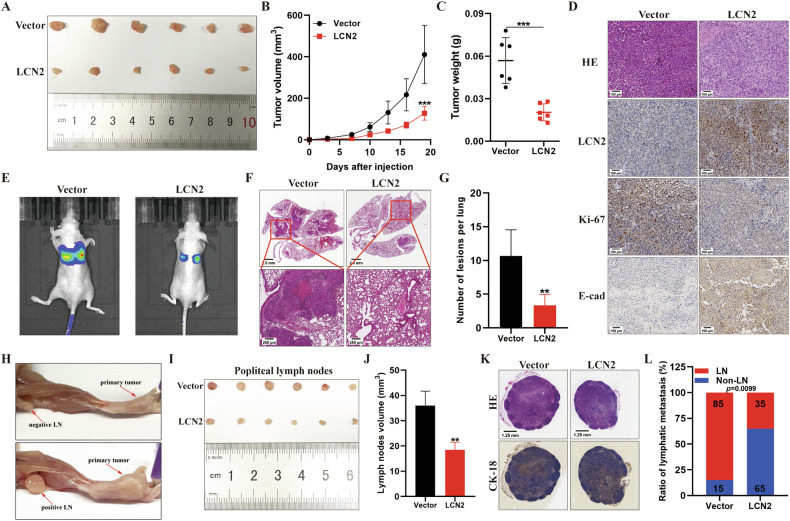
LCN2 suppresses GC tumor growth and metastasis in vivo. **A**–**C** Images of xenograft tumor samples (**A**), tumor volumes (**B**), and tumor weights (**C**) derived from MGC803-Vector and MGC803-LCN2 cells. **D** Representative images of HE staining and IHC staining for LCN2, Ki-67, and E-cadherin (*n* = 6), scale bar:100 μm. **E** Representative bioluminescence images of mice, 40 after tail vein injection of MGC803- Vector or MGC803-LCN2 cells. **F** Representative HE staining images of lung samples from (**E**). Scale bars: 2.5 mm (upper panel) and 250 μm (bottom panel). **G** Quantitative results of the metastatic nodes of the lungs in (**F**). **H** Representative images of popliteal lymph node metastasis and non-metastatic mice 32 days after footpad injection with the MGC803 cells. **I** Images of the popliteal lymph nodes of mice injected with the indicated cells. **J** Volume of the popliteal lymph nodes in (**I**). **K** Representative HE staining and CK-18 staining images of the popliteal lymph nodes in (**I**). Scale bars:1.25 mm (upper panel) and 100 μm (lower panel). **L** Percentage of metastatic and non-metastatic popliteal lymph nodes in the indicated groups. Data are presented as mean ± SD of three independent experiments. *p < 0.05, **p < 0.01, ***p < 0.001.

To confirm the results of in vitro analysis, the role of LCN2 in tumor growth and metastasis was investigated using a xenograft model. In nude mice, LCN2 overexpression in MGC803 cells (MGC-803-LCN2) reduced tumor volume and weight (Fig. 3A–C and Supplementary Fig. 3A). Furthermore, the number of Ki-67-positive cells significantly decreased in LCN2-overexpression group, while the number of E-cadherin-positive cells increased (Fig. 3D and Supplementary Fig. 3B). To investigate the effect of LCN2 on GC hematogenous metastasis in vivo, a lung metastasis model was established by injecting MGC803-vector or MGC803-LCN2 cells into the tail veins of nude mice. The LCN2 overexpression group exhibited significantly reduced metastatic nodules than the vector group at 40 days post-injection (Fig. 3E–G and Supplementary Fig. 3C). Furthermore, to determine the effect of LCN2 on LN metastasis of GC, an in vivo nude mouse popliteal LN metastasis model was used to simulate the directional drainage and metastasis of GC (Fig. 3H and Supplementary Fig. 3D). The volumes of LN were significantly smaller (Fig. 3I, J) and the lymphatic metastatic ratio was also lower in the LCN2 overexpression group than those in the vector group (Fig. 3K, L) at 32 days post-modeling. Collectively, these observations demonstrate that LCN2 also suppresses GC growth and metastasis in vivo.

### SPARC is a pivotal downstream target of LCN2 in GC

**Fig. 4:**
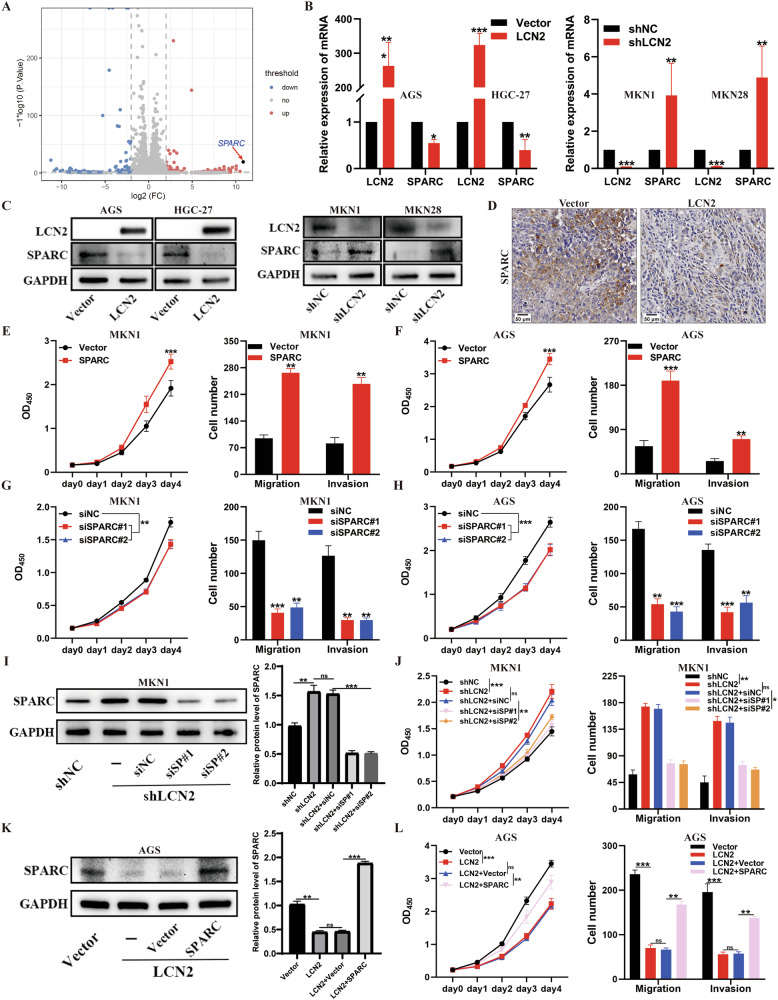
SPARC is a pivotal downstream target of LCN2 in GC. **A** Volcano plot showing the differential expression genes in negative control and LCN2 knockdown MKN1 cells. The x-axis represents the fold changes of read density and the y-axis shows the adjusted *p* value. **B**, **C** Detection of SPARC mRNA (**B**) and protein (**C**) levels in GC cells with LCN2 overexpression or knockdown using RT-qPCR and western blot. **D** IHC images of SPARC expression in xenograft tumor tissues with LCN2 overexpression and the negative control. Scale bar: 50 μm. **E**, **F** Promotion of the proliferation and metastasis ability of MKN1 (**E**) and AGS (**F**) cells by SPARC overexpression, as assessed by CCK-8 assay and transwell assay. **G**, **H** Inhibition of the proliferation and metastasis ability of MKN1 (**G**) and AGS (**H**) cells by SPARC knockdown, as assessed by CCK-8 assay and transwell assay. **I** Increase in SPARC protein level following LCN2 knockdown, which was rescued by SPARC siRNAs. Protein levels were measured by western blotting assay (left panel), and the grayscale was measured by ImageJ software. **J** CCK-8 and transwell assays showing that the proliferation and metastasis ability increased upon LCN2 knockdown, and this effect was rescued by SPARC knockdown. **K** Transfection of SPARC-overexpressed plasmids and the corresponding controls into LCN2-overexpressed AGS cells. Protein levels were measured by western blotting (left panel), and the grayscale was measured by ImageJ software. **L** CCK-8 and transwell assays revealed that SPARC overexpression rescued the suppression of proliferation and metastasis ability caused by LCN2 overexpression. Data are presented as mean ± SD of three independent experiments. *p < 0.05, **p < 0.01, ***p < 0.001.

To elucidate the molecular mechanism via which LCN2 inhibits GC growth and metastasis, we performed whole-genome expression profiling to analyze the effect of LCN2 knockdown on MKN1 cells. The results of RNA-seq showed that LCN2 knockdown promoted several oncogenic pathways, including MAPK and estrogen signaling pathways (Supplementary Fig. 4A). A volcano plot illustrated significant changes in gene expression following LCN2 knockdown (Fig. 4A). We identified 16 genes as pivotal downstream targets of LCN2 by setting gene changes ˃8 and *p-*values < 0.01 (Supplementary Fig. 4B). Next, we investigated the correlation between the expression levels of LCN2 and the 16 genes via TCGA database, and used qPCR to quantify the mRNA levels of these genes in LCN2-overexpressing cells, LCN2 knockdown cells, and the corresponding control cells (Supplementary Fig. 4C, D). The results indicated that SPARC may be a pivotal downstream target of LCN2 in GC (Fig. 4A). We also confirmed this finding using RT-qPCR analysis, in which LCN2 overexpression inhibited SPARC mRNA levels in AGS and HGC-27 cells, whereas LCN2 knockdown upregulated SPARC mRNA levels in MKN1 and MKN28 cells (Fig. 4B). Consistently, SPARC protein levels correlated negatively with LCN2 levels in GC cells (Fig. 4C). Moreover, IHC staining of the xenograft tumor samples also showed that LCN2 overexpression reduced SPARC protein levels (Fig. 4D). Subsequently, we assessed SPARC expression in GC cells using RT-qPCR and western blotting; results revealed high expression in AGS and HGC-27 cells but low expression in MKN1 and MKN28 cells (Supplementary Fig. 5A). Accordingly, we selected AGS and MKN1 cells for gain- or loss-of-function studies, and the efficiencies of stable overexpression and knockdown of SPARC were verified using RT-qPCR and western blotting (Supplementary Fig. 5B, C). The results showed that SPARC overexpression significantly promoted proliferation and metastasis in MKN1 and AGS cells (Fig. 4E, F and Supplementary Fig. 5D), whereas SPARC knockdown showed the opposite effect (Fig. 4G, H and Supplementary Fig. 5E). These results suggest that SPARC is a potential oncogene in GC and the pivotal downstream target of LCN2-rgegulated GC progression.

Furthermore, we performed a rescue experiment by targeting SPARC with siRNAs in LCN2-knockdown MKN1 cells and quantified SPARC protein levels using western blotting (Fig. 4I). We found that SPARC knockdown significantly inhibited the proliferation, migration, and invasion abilities of LCN2-knockdowned MKN1 cells (Fig. 4J and Supplementary Fig. 5F). Correspondingly, SPARC overexpression rescued the proliferation, migration, and invasion abilities of LCN2-overexpressed AGS cells (Fig. 4K, L and Supplementary Fig. 5G). These results all indicate that SPARC is a potential oncogene representing a crucial downstream target of LCN2 in GC.

### SPARC knockdown partially reverses GC progression induced by LCN2-knockdown in vivo

**Fig. 5:**
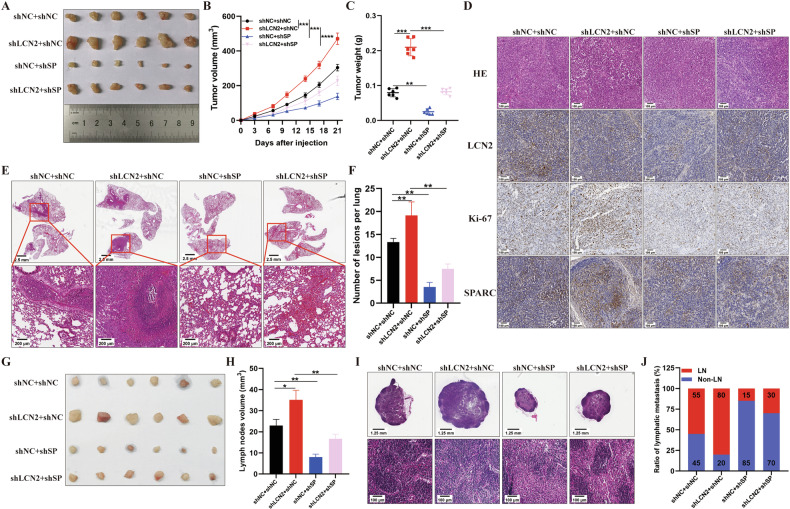
SPARC knockdown partially reverses GC progression induced by LCN2-knockdown in vivo. **A**–**C** Image of xenograft tumor samples (**A**), tumor volumes (**B**), and tumor weights (**C**) derived from mice injected with the indicated cells. **D** Representative images of HE staining and IHC staining for LCN2, Ki-67, and SPARC of each group (*n* = 6). Scale bar:100 μm; **E** Representative HE staining images of lung samples from the indicated groups. Scale bars:1.25 mm (upper panel) and 200 μm (lower panel). **F** Quantitative results of the metastatic nodes of the lungs in (**E**). **G** Images of the popliteal lymph nodes of mice injected with the indicated cells. **H** Volume of the popliteal lymph nodes in (**G**). **I** Representative HE staining images of the popliteal lymph nodes in (**G**). Scale bars:1.25 mm (upper panel) and 100 μm (lower panel). **J** Percentage of metastatic and non-metastatic popliteal lymph nodes in the indicated groups. Data are presented as mean ± SD of three independent experiments. *p < 0.05, **p < 0.01, ***p < 0.001.

To confirm that SPARC is a downstream target of LCN2 in vivo, we co-transfected MGC803 cells with LCN2 and SPARC knockdown lentiviruses. In a subcutaneous xenograft model, nude mice treated with MGC803-shLCN2 cells showed faster tumor progression in terms of volume and weight. However, tumor growth was reduced when SPARC was knocked down in MGC803 cells. Importantly, SPARC knockdown partially reversed the tumor-promoting effect induced by LCN2 knockdown (Fig. 5A–C and Supplementary Fig. 6A). Correspondingly, LCN2 knockdown promoted the expression of Ki-67 and SPARC; however, SPARC knockdown reversed the effect of LCN2 knockdown on Ki-67 expression (Fig. 5D and Supplementary Fig. 6B). Similarly, in vivo, lung and LN metastases were markedly increased in the LCN2-knockdown group, and further knockdown of SPARC partially restored these biological properties (Fig. 5E–J and Supplementary Fig. 6C, D). These results further reveal that LCN2 inhibits GC tumorigenicity and metastasis by downregulating the expression of SPARC.

### LCN2 downregulates SPARC expression by inhibiting the JNK/c-Jun signaling pathway

**Fig. 6:**
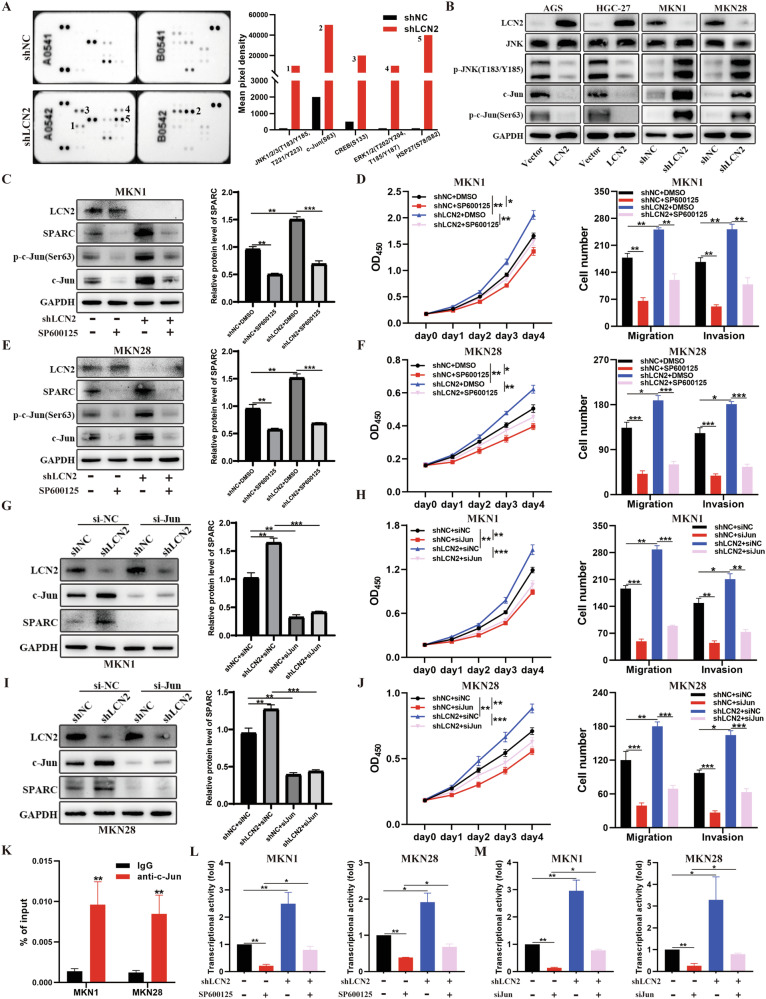
LCN2 downregulates SPARC expression by inhibiting the JNK/c-Jun signaling pathway. **A** Representative image of Phospho-proteomic profiling in negative control and LCN2 knockdown MKN1 cells (left panel) and quantitative results (right panel). **B** Detection of JNK, p-JNK, c-Jun, and p-c-Jun protein levels in GC cells with overexpression and knockdown of LCN2 using western blot. **C**, **E** Measurement of SPARC, c-Jun, and p-c-Jun protein levels in MKN1 (**C**) and MKN28 (**E**) cells combining with LCN2 knockdown and SP610025 treatment by western blotting (left panel), and grayscale measurement using ImageJ software. **D**, **F** CCK-8 and transwell assay showing that increased proliferation and metastasis ability upon LCN2 knockdown, which was rescued by SP610025 treatment. **G**, **I** Measurement of SPARC and c-Jun protein levels in MKN1 (**G**) and MKN28 (**I**) cells combining LCN2 knockdown and c-Jun knockdown using western blotting (left panel), and grayscale measurement using ImageJ software. **H**, **J** CCK-8 and transwell assay showing that increased proliferation and metastasis ability upon LCN2 knockdown, which was rescued by c-Jun knockdown. **K** ChIP-qPCR analysis demonstrating binding of c-Jun to the SPARC promoter region in MKN1 and MKN28 cells. **L**, **M** Assessment of SPARC promoter activity by combining LCN2 knockdown plasmid and SP610025 treatment (**L**) or c-Jun knockdown (**M**) using luciferase reporter assays in MKN1 and MKN28 cells. Data are presented as mean ± SD of three independent experiments. *P < 0.05, **P < 0.01, ***P < 0.001.

Next, we investigated the underlying mechanism of how LCN2 regulates SPARC expression. LCN2 could affect EMT process by regulating MMP protein expression in GC [15], and a close relationship between MMP protein expression and SPARC expression has also been found in other cancers [33]. Therefore, we examined SPARC expression after MMP2 or MMP9 overexpression in AGS cell, and found that MMP2 or MMP9 overexpression did not affect SPARC expression (Supplementary Fig. 7A). Given that LCN2 regulates SPARC expression independently of MMP protein in GC, we conducted a comprehensive analysis of RNA-seq data to identify the possible mechanism of action. LCN2 deficiency significantly altered the MAPK signaling pathway, which plays a crucial role in cancer progression. Further analysis using a phosphorylated protein array revealed significant upregulation of p-JNK (T183/Y185 and T221/Y223), p-c-Jun (Ser63), and ERK (T202/Y204 and T185/Y187) upon silencing of LCN2 in MKN1 cells (Fig. 6A). It has been reported that the amyloid precursor protein inhibits SPARC expression by inhibiting the transcriptional activity of c-Jun [34]. Furthermore, JASPAR database also indicated that there are potential binding sites for c-Jun in the promoter region of SPARC (Supplementary Tables 5 and 6). Therefore, we speculated that the JNK/c-Jun signaling pathway was involved in LCN2-regulated SPARC expression. Western blotting confirmed that LCN2 overexpression reduced p-JNK (T183/Y185 and T221/Y223), c-Jun, and p-c-Jun (Ser63) levels, while LCN2 knockdown increased the levels of these proteins (Fig. 6B). Meanwhile, we also found that LCN2 affected the levels of different phosphorylated forms of JNK (Supplementary Fig. 7B). Next, we used the JNK inhibitor, SP600125 (10 μM), to block the JNK/c-Jun signaling pathway in LCN2-knockdown MKN1 and MKN28 cells. LCN2 knockdown promoted SPARC expression, proliferation, and metastasis of GC cells, whereas SP600125 had an opposite effect. Blocking of the JNK/c-Jun signaling pathway dramatically inhibited the increase of the SPARC expression and the proliferation, migration, and invasion abilities of GC cells induced by LCN2 knockdown (Fig. 6C–F and Supplementary Fig. 7F). We also used siRNAs to target c-Jun in LCN2-knockdown MKN1 and MKN28 cells to further confirm that LCN2 regulated SPARC expression and GC progression in a JNK/c-Jun signaling pathway-dependent manner. Correspondingly, c-Jun knockdown also partially blocked the elevation in SPARC expression and the improvement in the proliferation, migration, and invasion abilities of LCN2-knockdown GC cells (Fig. 6G–J and Supplementary Fig. 7G). Overall, these findings suggest that the JNK/c-Jun signaling pathway plays a critical role in LCN2-regulated SPARC expression and GC progression.

Given that c-Jun and p-c-Jun acted as a transcription factor in colorectal cancer [34], breast cancer [35], and acute lymphoblastic leukemia [36], and the JASPAR database indicated the presence of c-Jun-binding sites in the promoter region of SPARC, we hypothesized that LCN2 inhibits the JNK/c-Jun signaling pathway and weakens the transcriptional activity of c-Jun and p-c-Jun to SPARC, ultimately leading to SPARC downregulation. To test this hypothesis, we firstly performed chromatin immunoprecipitation (ChIP) and determined whether c-Jun could bind to the promoter region of SPARC. The results showed that the c-Jun antibody significantly enriched in the SPARC promoter region, whereas the negative IgG did not show any such effect (Fig. 6K). Next, the luciferase reporter assays showed that the upregulated transcription activity of SPARC induced by LCN2 knockdown could be restored by the using of the JNK inhibitor (SP600125) or the siRNAs to target c-Jun in MKN1 and MKN28 cells (Fig. 6L, M). Furthermore, we also found that c-Jun knockdown significantly inhibited the SPARC expression, proliferation, migration, and invasion capabilities of GC cells (Supplementary Fig. 7C–E). Therefore, these results suggest that c-Jun and p-c-Jun act as transcription factors to induce SPARC expression, and LCN2 downregulates SPARC expression and inhibits GC progression via the JNK/c-Jun signaling pathway inhibition.

### LCN2 inhibits the activation of the JNK/c-Jun signaling pathway by binding to its receptor 24p3R

**Fig. 7:**
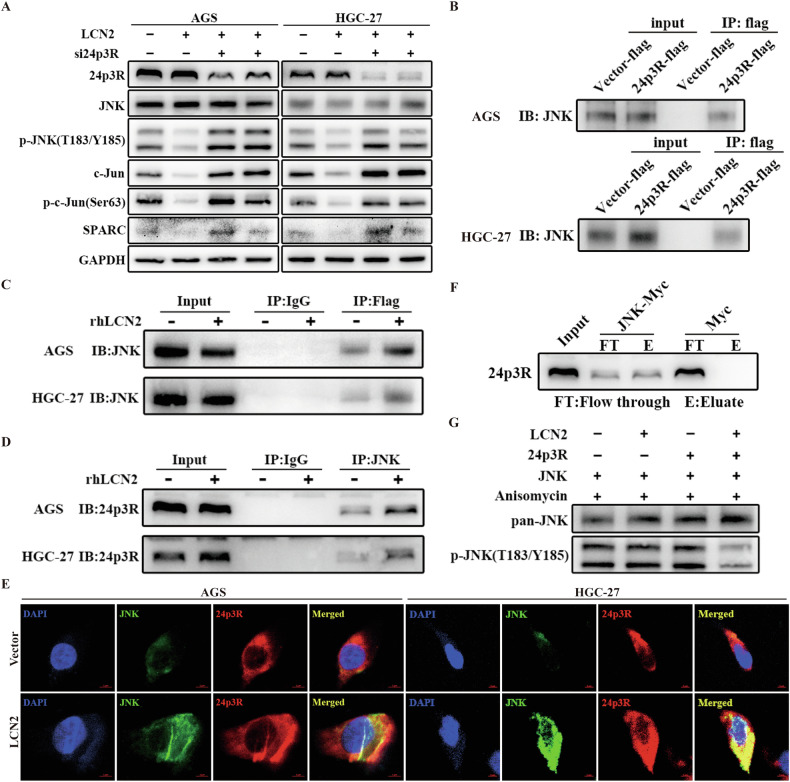
LCN2 inhibits the activation of the JNK/c-Jun signaling pathway by binding to its receptor 24p3R. **A** LCN2-overexperessed GC cells were transfected with siRNAs targeting 24p3R for 48 h, and then subjected to western blotting assays. **B**–**D** The whole cell lysate (B) and membrane fractions (**C**, **D**) of the indicated cells were subjected to co-immunoprecipitation and western blot to analyze the binding of JNK and 24p3R. **E** Immunofluorescence staining was used to detect the subcellular localization of JNK and 24p3R in negative control and LCN2 overexpression GC cells. **F** Magnetic beads linked to Myc-tagged JNK were incubated with purified recombinant 24p3R protein. The levels of 24p3R in the flow through and bound to the beads were detected by western blotting. **G** Recombinant LCN2 protein (40 μg/ml), 23p3R protein (40 μg/ml), JNK protein (200 μg/ml), and anisomycin were incubated for 30 min in kinase reaction buffer. The phosphorylated JNK level in the reaction was analyzed by western blotting. Data are presented as mean ± SD of three independent experiments. *p < 0.05, **p < 0.01, ***p < 0.001.

LCN2 reportedly regulates the signaling pathway by acting on 24p3R, the receptor for LCN2, in an autocrine manner [37]. We found that 24p3R inhibition could reverse the LCN2-induced decrease in p-JNK, c-Jun, p-c-Jun, and SPARC levels in GC cells, indicating that 24p3R was indispensable for LCN2-mediated inhibition of the JNK/c-Jun/SPARC axis activation (Fig. 7A). An immunoprecipitation assay revealed a possible binding between 24p3R and JNK (Fig. 7B–D). In addition, GST pull-down assays confirmed the direct interaction between 24p3R and JNK (Fig. 7F), and overexpression of LCN2 enhanced the binding of 24p3R and JNK to GC cell membranes (Fig. 7C–E). The in vitro JNK kinase assay showed that JNK was phosphorylated when incubated with anisomycin. However, this effect was significantly weakened when recombinant LCN2 and 24p3R proteins were present together (Fig. 7G), indicating that LCN2/24p3R signaling directly inhibited JNK phosphorylation. Collectively, these results demonstrate that LCN2 binds to 24p3R, and then downregulates SPARC expression by directly inhibits the activation of the JNK/c-Jun signaling pathway.

### Clinical relevance of LCN2, SPARC, and c-Jun in GC

**Fig. 8:**
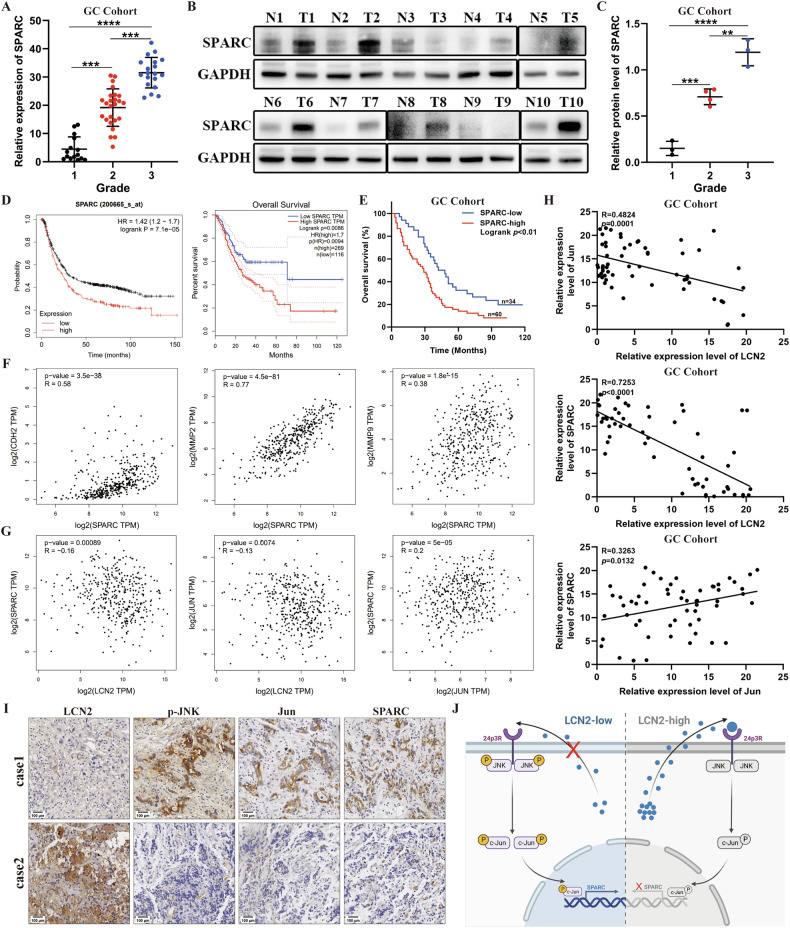
Clinical relevance of LCN2, SPARC, and c-Jun in GC patients. **A** SPARC mRNA expression was positively correlated with GC grade in our cohort (*n* = 59). **B** Detection of SPARC protein levels in GC tissues and their paired adjacent normal tissues using western blot (*n* = 10). **C** LCN2 protein expression was positively correlated with GC grade in our cohort. **D** TCGA and KM-plot database showing correlation between SPARC overexpression and poorer prognosis. **E** Kaplan–Meier survival analysis revealing shorter overall survival times in patients with high SPARC expression in our cohort (*n* = 94, *p* < 0.001, log-rank test). **F** Correlation analysis between SPARC expression and CDH2, MMP2, and MMP9 expression in GC using the TCGA database. **G** Correlation analysis of LCN2 expression, SPARC expression and c-Jun expression in GC using the TCGA database. **H** Correlation analysis of LCN2, SPARC, and c-Jun mRNA expression in our cohort using RT-qPCR. **I** Negative correlation between LCN2 levels and p-JNK, c-Jun, and SPARC levels in GC specimens. Scale bars: 100 μm. **J** Illustrative model showing the proposed mechanism by which Tumor-secreted LCN2 impairs gastric cancer progression through autocrine inhibition of the 24p3R/JNK/c-Jun/SPARC axis. Data are presented as mean ± SD of three independent experiments. *p < 0.05, **p < 0.01, ***p < 0.001.

Finally, we assessed whether the LCN2/JNK/c-Jun/SPARC axis identified in vitro was clinically relevant to GC. We found that SPARC was significantly upregulated in GC tissues compared to that in paired adjacent non-tumor tissues, and that its expression levels correlated positively with higher tumor grade in patients with GC from TCGA cohort (Supplementary Fig. 8A). Consistently, RT-qPCR and western blotting in our cohort showed significant overexpression of SPARC and c-Jun in GC tissues compared to that in paired non-tumor tissues, and a positive correlation was observed between SPARC expression and GC grade (Fig. 8A–C and Supplementary Fig. 8B, C). Moreover, SPARC and c-Jun overexpression were associated with shorter OS in patients with GC in TCGA database and our cohort (Fig. 8D–E and Supplementary Fig. 8D). Moreover, we also observed a positive correlation between the expression of SPARC and N-cadherin, MMP2, and MMP9 in GC (Fig. 8F). Furthermore, analysis of LCN2, c-Jun, and SPARC expression levels in GC tissues from TCGA and our cohort revealed a negative correlation between LCN2 and c-Jun and SPARC expression levels, and a positive correlation between c-Jun and SPARC expression (Fig. 8G–H). IHC also revealed that lower LCN2 expression corresponded with higher levels of p-JNK, c-Jun, and SPARC in GC tissues (Fig. 8I). Taken together, these findings demonstrate that the LCN2/JNK/c-Jun/SPARC axis is widely involved in the growth and metastasis of GC.

## Discussion

Abnormal expression of oncogenes or suppressor genes is closely implicated in tumor occurrence and development. Here, we elucidated the inhibitory role of LCN2 in GC progression and describe a novel mechanism of LCN2-regulated GC progression using comprehensive clinical sample, in vitro, and in vivo data. LCN2, which belongs to the LCN family of proteins involved in various cellular functions [38], has been extensively studied and is closely linked to tumor development [7]. LCN2 is overexpressed in various cancers and has been identified as a potential therapeutic target [10–13]. However, the role of LCN2 as an oncoprotein or suppressor protein is still debated. For instance, few studies reported that LCN2 could promote cancer growth while others suggested LCN2 as a tumor suppressor [14, 39–41]. Besides, current studies on the role of LCN2 in tumors have mainly focused on its function as an intracellular protein. Kubben et al. found that increased levels of MMP-9/lipocalin-2 complexes in GC tissues were associated with reduced survival [42]. However, the basic form of LCN2 is a secreted protein, and a recent study also reported the role and mechanism of LCN2 as secreted protein in tumor progression. In this study, we demonstrated that LCN2 was aberrantly expressed in GC and negatively correlated with tumor grade and poor prognosis in patients with GC by combining the results of our previous scRNA-seq analysis data with information from database and our clinical cohort. The specific mechanism of LCN2 as a secreted protein regulating GC progression were described and confirmed. To the best of our knowledge, this is the first study to determine the role of LCN2 in GC progression by synthesizing multiple data, and also the first to reveal the mechanism of LCN2 as a secreted protein regulating GC progression.

Substantial evidence suggests that the occurrence and development of GC is related to the aberrant of various intracellular signaling pathways. Moreover, LCN2 has been found to influence tumor progression by regulating various intracellular signaling pathways [11, 40]. More importantly, a recent study showed that the secreted protein, LCN2, binds to its receptor 24p3R, which directly phosphorylates JAK2 and activates the JAK2/STAT3 pathway, ultimately leading to produce increased amounts of an angiogenic factor, CXCL1 [37]. Here, our GSEA analysis and phosphorylation sequencing revealed that LCN2 expression was strongly associated with the JNK/c-Jun pathway. The JNK/c-Jun pathway is a critical intracellular pathway, which is widely involved in the regulation of cell proliferation, migration, and cancer progression [43]. The aberrant activation of the JNK/c-Jun pathway has been linked to the development of multiple cancers, including GC [44–46]. In this study, the results of RNA-seq and phosphokinase array indicated that JNK/c-Jun pathway activation was negatively correlated with the LCN2 expression. Blocking the JNK/c-Jun pathway could largely rescue the promotion effect of knocking down LCN2 on proliferation and metastasis in GC cells. More importantly, we revealed the precise mechanisms of how the secreted protein, LCN2, binds to its receptor 24p3R via autocrine, and then directly inhibit the JNK/c-Jun pathway activation. Taken together, our study suggests that LCN2 negatively regulates the JNK/c-Jun signaling pathway to exert its tumor-inhibitory functions in GC.

Both unphosphorylated c-Jun and phosphorylated c-Jun could promote tumor progression by regulating the transcription of many important cell-proliferating and growth-regulating genes. The JNK family of MAP kinase phosphorylates c-Jun at Ser-63, thus greatly enhancing the transcriptional activity of c-Jun. Here, we identified that SPARC as a key target of the LCN2/24p3R/JNK/c-Jun axis, and the transcription factors, c-Jun and p-c-Jun, activate the transcription of SPARC via directly binding to the promoter region of SPARC. SPARC belongs to the matricellular family of secreted proteins that plays a critical role in mediating cell–matrix interactions [47]. The role of SPARC in cell survival and death remains controversial and appears to be cell-type specific. In most studies, SPARC has been reported as an oncogene [25, 30, 48]. These studies are consistent with our results that SPARC overexpression in GC cells shortens cell population doubling time, enhances cell migration ability, and increases tumor cell invasiveness. Contrary to these observations, SPARC can also delay tumor growth and metastasis in certain cancer types [49, 50]. These phenomena suggest that SPARC plays different roles in tumor progression across different tumor cell types and acts via different signal transduction pathways. In this study, SPARC upregulation in GC tissues significantly correlated with poor patient prognosis. SPARC knockdown significantly inhibited GC cell proliferation and metastasis, and GC cells with LCN2 knockdown showed markedly reversed cell proliferation and metastasis after SPARC siRNA transfection. Furthermore, we demonstrated that LCN2 affects SPARC expression by regulating the transcriptional activity of c-Jun and p-c-Jun. Collectively, these findings suggest that SPARC is a pivotal downstream target of the LCN2/24p3R/JNK/c-Jun axis, and is also a necessary and sufficient key oncogenic driver of GC progression.

In summary, our study highlights the crucial role of LCN2 in inhibiting GC progression. LCN2 downregulates SPARC expression by inhibiting c-Jun and p-c-Jun transcriptional activity, thereby affecting GC growth and metastasis. Furthermore, LCN2 binds to the receptor, 24p3R, on GC cell surfaces via autocrine, directly inhibiting the JNK/c-Jun signaling pathway. Clinically, LCN2 expression in GC negatively correlates with tumor grade, poor prognosis, SPARC expression, and c-Jun expression. Therefore, targeting of the LCN2/24p3R/JNK/c-Jun/SPARC axis may be an effective strategy to improve the survival rate of patients with GC.

## Materials and methods

### Clinical samples

A tissue microarray containing 107 primary GC tissues with comparable clinicopathological features and follow-up data (13 of these patients were lost to follow-up) was used for IHC and prognostic analysis. In addition, a second cohort comprising 59 paired fresh tumor and adjacent non-tumor tissues was used for RT-qPCR and partially for western blotting. Furthermore, a third cohort comprising 50 paired tumors and adjacent non-tumor tissues was used for IHC. All samples were collected at the First Affiliated Hospital of Sun Yat-sen University. This study was approved by the Ethics Committee of the First Affiliated Hospital of Sun Yat-sen University (project number 2020-164), and written informed consent was obtained from each patient before sample collection.

### Cell lines, cell culture, and reagents

AGS, MKN1, MKN28, HGC-27, MGC803, and GES-1 cell lines were obtained from the American Type Culture Collection (ATCC). AGS and MGC803 cells were cultured in Dulbecco’s modified Eagle’s medium (Gibco, Waltham, MA, USA), while MKN1, MKN28, HGC-27 and GES-1 cells were cultured in Roswell Park Memorial Institute-1640 medium (Gibco) supplemented with 10% fetal bovine serum (FBS, Gibco) and 1% penicillin/streptomycin. All cells were incubated in a 5% CO_2_ incubator at 37 °C and routinely checked for Mycoplasma infection using the Mycoplasma detection kit (Beyotime, Guangzhou, Guangdong, China).

Drug treatments, including those with SP600125 (HY-12041; MCE, Shanghai, China) and anisomycin (HY-18982; MCE), were performed as described previously [51, 52]. Briefly, cells were seeded in a 6-well plate, and 10 μmol/l SP600125 was added to the medium when the cell density reached 70–80%.

### RNA interference and lentivirus transduction

To generate stable overexpression and knockdown in GC cells, lentiviruses were constructed by iGene Biotechnology (Guangzhou, Guangdong, China). In brief, lentiviruses were added to the culture medium at a multiplicity of index of 1:100, and the fresh medium was replaced after 48 h. Then, the infected cells were incubated with 2 μg/ml puromycin for 2 weeks to screen for the stably transfected cells. The target sequences of siRNA were synthesized and purified by JIJIE Biotechnology (Guangzhou, Guangdong, China), as listed in Supplementary Table 2. siRNA transfections were performed using Lipofectamine RNA iMAX reagent (Invitrogen, Carlsbad, CA, USA) according to the manufacturer’s instructions.

### CCK-8 proliferation and colony formation assays

Cell proliferation was assessed using the CCK-8 assay (Boster Biological Technology, Wuhan, Hubei, China) according to the manufacturer’s instructions. Briefly, six replicates of GC cells were seeded (1000 cells per well) in 96-well plates and cultured in 200 µl medium. Then, 10 μl CCK-8 was added into each well at the same time and incubated in a humidified incubator for 3 h at 37 °C in the presence of 5% CO_2_. Finally, the absorbance was measured at 450 nm. For colony formation assays, 500 cells per well were seeded in a 6-well plate and the medium was changed twice per week. After 20 days, the colonies were fixed using 4% paraformaldehyde for 30 min at room temperature and then stained using 0.5% crystal violet for 20 min. Finally, the number of colonies was photographed and counted.

### Transwell cell migration and invasion assay

Cell migration and invasion were examined using transwell chambers (8 μm inserts; Corning, Tewksbury, MA, USA) in a 24-well plate. For migration assays, 5 × 10^4^ GC cells suspended in 300 μl serum-free medium were seeded into the upper chambers, while the lower chambers were filed with 600 μl culture medium containing 10% FBS. After 24−48 h, the cells were fixed, stained, and randomly imaged using a microscope (Olympus, Tokyo, Japan). For the invasion assays, 5 × 10^4^ GC cells were inoculated in the upper chamber of Matrigel-coated inserts (Corning), and the other steps followed were the same as that in migration assays.

### Wound healing assay

The GC cells were seeded into a 6-well culture plate and cultured at 37 °C in the presence of 5% CO_2_. The medium was removed when the cells reached 90% confluence and the surface of the inoculated cells was scratched using a 20 μl pipette tip, followed by gentle washing using PBS. Two milliliters of serum-free medium were added to continue the culture. Finally, the scratches were photographed at 0 h and 24 h.

### RNA isolation and RT-qPCR

Total RNA was isolated from tissues and cells using the Trizol reagent (Takara, Beijing, China) according to the manufacturer’s protocol. RNA was subjected to cDNA synthesis using the master mix cDNA synthesis kit (Accurate Biotechnology, Changsha, Hunan, China). qPCR was performed using SYBR Green (Accurate Biotechnology) in a LightCycle480 II platform (Roche, Switzerland). mRNA expression was analyzed using the 2^-ΔΔCt^ method and normalized to *GAPDH* expression, which was used as an internal control. The primers used in this study are listed in Supplementary Table 3.

### Protein extraction and western blotting

For protein extraction, tissues and cells were lysed in radioimmunoprecipitation assay buffer (Beyotime) and then centrifuged at 12,000 *×* *g* at 4 °C for 15 min. For western blotting, the protein lysates were separated using sodium dodecyl sulfate-polyacrylamide gel electrophoresis and transferred to polyvinylidene difluoride membranes. The membranes were incubated with primary antibodies overnight at 4 °C, washed, and then probed with horse radish peroxidase-conjugated secondary antibodies at room temperature for 1 h. Finally, the chemiluminescence signals were visualized using enhanced chemiluminescence reagent. All the antibodies used in this study are listed in Supplementary Table 4.

### Phospho-proteomic profiling

LCN2-knockdown MKN1 cells and control cells were washed thrice with phosphate-buffered saline (PBS) and resuspended in lysis buffer. The cell lysates were then centrifuged at 14,000 × *g* for 5 min, and the supernatant was transferred into a clean test tube. After quantifying the protein concentration using the bicinchoninic acid assay kit (Beyotime), cell lysates containing 500 µg total protein were diluted and detected using the proteome profiler human phospho-kinase array (R&D Systems, Minneapolis, MN, USA) following the manufacturer’s instructions.

### ChIP

ChIP assays with MKN1 and MKN28 cells were performed using a ChIP assay kit (Cell Signaling Technology, MA, Boston, USA), following the manufacturer’s protocol. The cells were crosslinked with 1% paraformaldehyde for 15 min at room temperature, followed by glycine quenching. After washing with PBS, the cells were centrifuged and resuspended in lysis buffer. The fragmented chromatin was incubated with anti-IgG and anti-c-Jun and then incubated with protein A/G magnetic beads at 4 °C for 2 h. Finally, the immunoprecipitated DNA was purified and analyzed using qPCR with the primers listed in Supplementary Table 3.

### Dual-luciferase reporter assay

The SPARC promoter region was amplified and subcloned into the GV-148 vector (GeneChem, Shanghai, China) for the luciferase assay. Cells were seeded in 24-well plates at a density of 10,000 cells/well. After overnight incubation, the cells were transfected with the corresponding plasmids using Lipofectamine 3000 (Invitrogen). Finally, the luciferase and renilla signals were measured 48 h after transfection using a dual-luciferase reporter assay kit (Beyotime) following the manufacturer’s instructions.

### Co-immunoprecipitation (Co-IP)

Cell lysates were prepared by lysing AGS and HGC-27 cells using a cell lysis buffer (Beyotime). The lysates containing 5 mg protein were incubated with a specific primary antibody overnight at 4 C. Then, protein A/G agarose (Santa Cruz Biotechnology, Santa Cruz, CA, USA) was incubated with the obtained protein complex for 2 h at 4 °C. Finally, the immune-precipitates were washed and analyzed using western blotting.

### JNK-activated protein kinase assay

For the kinase reaction, JNK, anisomycin, LCN2, and 24p3R were combined in a kinase reaction buffer (25 mM Tris, pH 7.4, 0.5 mM sodium vanadate, 20 mM MgCl_2_, 2 mM MnCl_2_, 20 mM ATP) on ice. After 30 min of incubation at 30 °C, sodium dodecyl sulfate sample buffer was added to terminate the reactions. The samples were resolved and subjected to western blot analysis.

### IHC

Paraffin-embedded sections were deparaffinized using xylene, followed by antigen retrieval using an autoclave in 0.01 mol/l EDTA buffer (pH 8.0). The sections were incubated with primary antibodies overnight at 4 °C. The proportion of positively stained cells was scored as follows: 0, 0%; 1, <10%; 2, 10–40%; 3, 40–75%; and 4, >75%. Staining intensity was scored from 0 to 3 (0, no staining; 1, weak staining; 2, moderate staining; 3, strong staining). The scores for the percentage of positive cells and staining intensity were multiplied to calculate the immunoreactivity score (IRS; range, 0–12) for each sample. IRS score <6 was defined as low expression and the rest as high expression.

### Animal studies

For the xenograft model, 4–6-week-old female BALB/c nude mice were purchased from SPF Biotechnology Co., Ltd. (Beijing, China). All mice were housed in a specific pathogen-free facility and kept 12 h of light/12 h of darkness. Besides, mice were randomly assigned to each group (6 mice per group). The animal experiments were approved by the Animal Care and Use Committee of Sun Yat-sen University (No. 2021019) and conducted in accordance with the regulations and operational procedures of experimental animal management. MGC803 cells (5 × 10^6^) resuspended in 100 μl PBS were subcutaneously injected into the right flank of the mice. Tumor volume was measured every 3 days and calculated using the formula: (length × width^2^)/2. The mice were sacrificed 21 days after tumor cell implantation. To assess lung metastasis, MGC803 cells (1 × 10^6^ in 100 μl PBS) were injected into the tail vein of 6-week-old female BALB/c nude mice. All mice were sacrificed 8 weeks after injection. Intact lungs were resected, photographed, and fixed in 4% paraformaldehyde for further analysis. Metastatic nodules in the lungs were counted using a digital microscope (Nikon, Tokyo, Japan). For the popliteal LN metastasis model, a 30 μl suspension containing 1 × 10^6^ MGC803 cells was implanted into the footpads of mice. The primary footpad tumors and popliteal LNs were resected, photographed, fixed in 4% paraformaldehyde, and subjected to IHC once the tumor volume reached ~500 mm^3^. In all animal studies, we used a blind approach to reduce biases in data collection and analysis. Researchers are not aware of specific groups when conducting and evaluating experiments.

### Statistical analysis

Statistical analysis was performed using the GraphPad Prism 8 software (GraphPad, La Jolla, CA, USA). The independent T-test was used for comparison between two groups and one-way analysis of variance was used for comparison within multiple groups. A two-sided statistical test was consistently conducted, with a minimum sample size of *n* ≥ 3 was considered. The log-rank test was applied to examine potential survival differences among two or more groups in the Kaplan–Meier analysis. *p* value < 0.05 was considered statistically significant (*p < 0.05, **p < 0.01, and ***p < 0.001).

## Supplementary information

Supplementary Information

Supplementary Table 7-RNA-seq data about shNC and shLCN2

Original western blots

## Data Availability

The data that support the findings of this study are available from the corresponding author upon reasonable request.
