# Supplementary material for: Tumor-secreted LCN2 impairs gastric cancer progression via autocrine inhibition of the 24p3R/JNK/c-Jun/SPARC axis
Source: Cell Death Dis. 2024 Oct 18;15(10):756. doi: 10.1038/s41419-024-07153-z (PMC11489581; doi:10.1038/s41419-024-07153-z)
Supplement: Supplementary file 1 — Supplementary Information [file 41419_2024_7153_MOESM1_ESM.docx]

**Supplementary Tables**

**Supplementary Table 1. Correlation between LCN2 expression and clinical characteristics**

|  | Total (n=144) | LCN2-low (n=80) LCN2-high (n=64) | | *p* |
| --- | --- | --- | --- | --- |
| Age 0.9614 | | | | |
| <60 | 54 | 30 | 24 |  |
| ≥60 | 90 | 50 | 40 |  |
| Gender 0.4554 | | | | |
| Male | 68 | 40 | 28 |  |
| Female | 76 | 40 | 36 |  |
| Pathological type 0.1336 | | | | |
| adenocarcinoma | 120 | 70 | 50 |  |
| others | 15 | 10 | 14 |  |
| T stage 0.0038 | | | | |
| T1-2 | 45 | 17 | 28 |  |
| T3-4 | 99 | 63 | 36 |  |
| Distant metastasis 0.0139 | | | | |
| M0 | 102 | 50 | 52 |  |
| M1 | 42 | 30 | 12 |  |
| LN-metastasis 0.0020 | | | | |
| N0 | 48 | 18 | 30 |  |
| N1-3 | 96 | 62 | 34 |  |

**Supplementary Table 2. Sequences of shRNA and siRNA used in this study.**

| Gene name | sequences |
| --- | --- |
| SPARC#1 sense  SPARC#1 antisense  SPARC#2 sense  SPARC#2 antisense  c-Jun#1 sense  c-Jun#1 antisense  c-Jun#2 sense  c-Jun#2 antisense  c-Jun#3 sense  c-Jun#3 antisense  24p3R#1 sense  24p3R#1 antisense  24p3R#2 sense  24p3R#2 antisense | GGUGCUAACAUAGAUUUAA dTdT  TTAAATCTATGTTAGCACC dTdT  GCUGCUUCUUAUUAAUCAU dTdT  ATGATTAATAAGAAGCAGC dTdT  GCGGGAGGCAUCUUAAUUA dTdT  TAATTAAGATGCCTCCCGC dTdT  GUGCGCUCUUAGAGAAACU dTdT  AGTTTCTCTAAGAGCGCAC dTdT  CAGUGCUUCUUACUAUUAA dTdT  TTAATAGTAAGAAGCACTG dTdT  GCCUGUGGGAUUAUCUGAA dTdT  UGAAACAGAAUGAUAGAGC dTdT  GCCUGUGGGAUUAUCUGAA dTdT  UUCAGAUAAUCCCACAGGC dTdT |

**Supplementary Table 3. Sequences of primers used in this study.**

| Primer name | Primer sequences |
| --- | --- |
| Primers for real-time PCR:  GAPDH-F  GAPDH-R  LCN2-F  LCN2-R  SPARC-F  SPARC-R  c-Jun-F  c-Jun-R  UCHL1-F  UCHL1-R  ZNF709-F  ZNF709-R  QKI-F  QKI-R  NPY1R-F  NPY1R-R  PTPRR-F  PTPRR-R  CTAGE8-F  CTAGE8-R  TMEM265-F  TMEM265-R | 5'-AGCCACATCGCTCAGACAC-3'  5'-GCCCAATACGACCAAATCC-3'  5'- GAGTTACCCTGGATTAACGAGT -3'  5'- AAGCGGATGAAGTTCTCCTTTA -3'  5'-CCCTGTACACTGGCAGTTCG-3'  5'-CCAGGGCGATGTACTTGTCA-3'  5'-GAAGTAGCCCCCAACCTCTC-3'  5'-ATGGCTCTCAACTCAAGCGT-3'  5'- CTTCATGAAGCAGACCATTG-3'  5'- ATCATGGGCTGCCTGTATG-3'  5'- CAGTTTGGAGCACCATATC-3'  5'- AGAGATAAATGGCCAATGA-3'  5'- ATTATTGGTACCTGCAGCAG-3'  5'- TAGGTGCCATTCAGAATCG-3'  5'- TATACCACTCTTCTCTTGGTGCTG-3'  5'- CTGGAAGTTTTTGTTCAGGAACCCA-3'  5'- ACCTATCGCCCATCACATTACA-3'  5'- GCGGTGGTAGCTTTGATCTCA-3'  5'- GGTCCTGCGCATTCATCTTC-3'  5'- GCCTGTGATGGCACTCTGAA-3'  5'- CGCGGCTGGCAAGTGA-3'  5'-ACTTTCTGATAACGATGACACCTGA-3' |
| Primers used for ChIP-qPCR: | |
| SPARC promoter-F | 5'-CCTCCCAGAGTGTTAGGCAC-3' |
| SPARC promoter-R | 5'-CCAGGCCCAAGTGTCTTGAT-3' |

**Supplementary Table 4. Antibodies used in this study for WB, ChIP, IHC and IF.**

| Antibody | Application | Dilution | Supplier | Catalog number |
| --- | --- | --- | --- | --- |
| GAPDH | WB | 1/5000 | Proteintech | Cat No. 60004-1-Ig |
| LCN2 | WB | 1/1000 | Abcam | ab125075 |
| LCN2 | IHC | 1/200 | Proteintech | Cat No. 26991-1-AP |
| SPARC | WB | 1/1000 | Abcam | ab207743 |
| SPARC | IHC | 1/200 | Proteintech | Cat No. 15274-1-AP |
| JNK | WB | 1/1000 | CST | #9252 |
| JNK | Co-IP | 1:30 | Proteintech | Cat No. 24164-1-AP |
| JNK | IF | 1:100 | Proteintech | Cat No. 66210-1-Ig |
| p-JNK(T183/Y185) | WB  IHC | WB: 1/1000  IHC: 1/250 | CST | #4668 |
| c-Jun | WB  ChIP | WB: 1/1000  ChIP: 1/50 | CST | #9165 |
| p-c-Jun(ser73) | WB | 1/1000 | CST | #3270 |
| 24p3R | WB  IF | WB: 1/1000  IF: 1/100 | Abcam | ab237539 |
| Flag | Co-IP | 1:30 | Abcam | ab205606 |
| Rabbit IgG | ChIP | ChIP: 1/50 | CST | #2729 |
| Ki-67 | IHC | 1/500 | CST | #9027 |
| E-cad | IHC | 1/250 | Proteintech | Cat No. 20874-1-AP |
| Mouse IgG | WB | 1/5000 | DIA-AN | Q6004 |
| Rabbit IgG | WB | 1/5000 | CST | #7074 |
| IHC mouse/rabbit antibody | IHC | - | Servicebio | G1210-2-A |

Abbreviations: WB, western blotting; IHC, Immunohistochemistry; ChIP, Chromatin immunoprecipitation; IF, immunofluorescence.

**Supplementary Table 5. The promoter region of SPARC**

GTTCGAGACCAGTCTGGCCAACATGGTGAAACCCTGTCTCTACTAAAAATACAAAAATTAGCCTGGTGTGGTGGTGCATGCCTGTAATCCCAGCTTCTTGAGAGGCTGAGGCAGCAGAATCACTTGAACCCAGGAGGTGGAGGTTGCAGTGAGTTGAGATTGCGCCACAGCCCTCCAGCCTGGGCAACAAAGTGAGAGTCTGTCTCAAAAAAAAAAAAAAAATCCTGGGGTGGGAGAAGCTTTGGGGTAGTTGAAGGACTGGAAGGAAGTTGGCATGGCTGGAGCAGCGTGGGCACAGAGGACAGTGGTAGGAGGAGAGAATGGCAAGAGAGACAGGGGCCACAGCGGATGGAAGTGTATCATGAGGGTGTTCTCTGCCTTCTTCCTTTCCATGGTATTCAAACCCAACCCATGCTTCCAGGTCCACCTCTTTCAGGAATCCACCAATCTGAAGCCCTGGGATAAGCCTTAGAGTCTGTACCACTCATGTAAGTGGGTGTATGTGTAAGGCACAACCTTTCAGCAAGATCCAAAGCTCCTTGAATAGTGAGTTATCTCGATTGATTGTTCACAGTCACAGATTGAATTCCTTGTACTTTTTTTCCCTTCTCAGTTCTGCACTTAACTCGTCTAAAAAAATTAAAAAAGAATTTAAGAAACCACAAAGCTAAGCTGGGTGCGGTGGCTCACGCCTGTAATCCTAGCACTTTGGGAAGCCAAGGCATTCGGATTGCCCAAGCTCAGGAGTTCGAGACCAGCCTGGGCAACATGTTGAAACCCCATTTCTACTAAAAATACAATAAATTAGCTGGGTGTTGTGGCATGTGCGCCTGTAATCCCAGCTACTCTGGAGGCTGAGGCGCGATAATTGCTTGAACCCGGGAGGCAGAGGTTGCAGTGAGCCGAAATCATACCACTGCACTCCAGCCTGGGCGACAGAGTGAGTGAGACTCTGTCTCAAAACAAAACAAAACAAACAAACAAAAAAACCGGAAACCACAAAACTTTTTGAGGACAAGGACCAGGTATTTATTAATTCTCATACCTCCCAGAGTGTTAGGCACAAAATAAACATTCAACCAAGACCTGTTGCACTGAGCAGTTCATATATAACAGGAGTGACCCAAGTTGAAACGTAGAATCAGCCCTCTCATACCACTTTTTGCCAGGTGATCATAGGCAAGTTACTTAGCATCTATGTTTCCTTATTATTAAAATGGTCATAATTACAATGCCTAAGATAAGGGGTTGCTGTGAAGATTATTAAATCCTCAGTAAACTTTGGCTATTGTTACTCCTATGATTATCATCAATATCATCAATTACCTTATCTGTTCAATACTGGTGGCACAGGTCCACCAGCTAGATGTCTAATCCCTTATGTGTCTATTAGTGGTACAAGTGGAGTTTGAGTGGGATTTTTTTTTTTAAGACCAGTTCCAAATCATCAAGGATGATACCACTAGTAGCAGCTTGTCTTGTCTGTACAGTGGTAAGTCCTGGCCTTGCCTTTGTGGCAAATACAACCCCCTTGAATTGCTTGGCCCTTCTCAGCATTGCCTAATATTAGGGAGGACTCCTGTAAAGCTCACTGGTTAGAAGATCAAGACACTTGGGCCTGGTTCTGCCCCTGGGGGCCATTGGGTAATTCCTTGCAGTCTCCAGGCCTCACTTGCCCTCTGAACAAGAAAGAGGCTGTTCTGGGTCATCCCTCCAGGCCTGTCCAGCCCTGGCACTCTGTGAGTCGGTTTAGGCAGCAGCCCCGGAACAGATGAGGCAGGCAGGGTTGGGACGTTTGGTCAGGACAGCCCACCGCAAAAAGAGGAGGAAAGAAATGAAAGACAGAGACAGCTTTGGCTATGGGAGAAGGAGGAGGCCGGGGGAAGGAGGAGACAGGAGGAGGAGGGACCACGGGGTGGAGGGGAGATAGACCCAGCCCAGAGCTCTGAGTGGTTTCCTGTTGCCTGTCTCTAAACCCCTCCACATTCCCGCGGTCCTTCagactgcccggagagcgcgctctgcctgccgcctgcctgcctgccactgag

**Supplementary Table 6. JASPAR analysis of c-Jun binding sites in promotor regions of SPARC.**

| **Matrix ID** | **Score** | **Relative score** | **Start** | **End** | **Strand** | **Predicted sequence** |
| --- | --- | --- | --- | --- | --- | --- |
| [MA0489.1](https://jaspar.genereg.net/matrix/MA0489.1) | 7.364807 | 0.8597060139905 | 1701 | 1714 | - | GAGGGATGACCCAG |
| [MA0489.1](https://jaspar.genereg.net/matrix/MA0489.1) | 6.430609 | 0.8476846498442 | 542 | 555 | + | GAATAGTGAGTTAT |
| [MA0488.1](https://jaspar.genereg.net/matrix/MA0488.1) | 6.243372 | 0.8557171571224 | 1322 | 1334 | - | CAGATAAGGTAAT |
| [MA0489.1](https://jaspar.genereg.net/matrix/MA0489.1) | 6.091105 | 0.8433158709057 | 1767 | 1780 | + | AACAGATGAGGCAG |
| [MA0488.1](https://jaspar.genereg.net/matrix/MA0488.1) | 5.9293194 | 0.8522967797556 | 1556 | 1568 | - | AATATTAGGCAAT |
| [MA0489.1](https://jaspar.genereg.net/matrix/MA0489.1) | 5.592018 | 0.8368935648509 | 547 | 560 | - | TCGAGATAACTCAC |
| [MA0488.1](https://jaspar.genereg.net/matrix/MA0488.1) | 5.5258694 | 0.8479027662356 | 1451 | 1463 | + | AGGATGATACCAC |
| [MA0489.1](https://jaspar.genereg.net/matrix/MA0489.1) | 5.429485 | 0.8348020682012 | 936 | 949 | + | ACAGAGTGAGTGAG |

**Supplementary Figures**


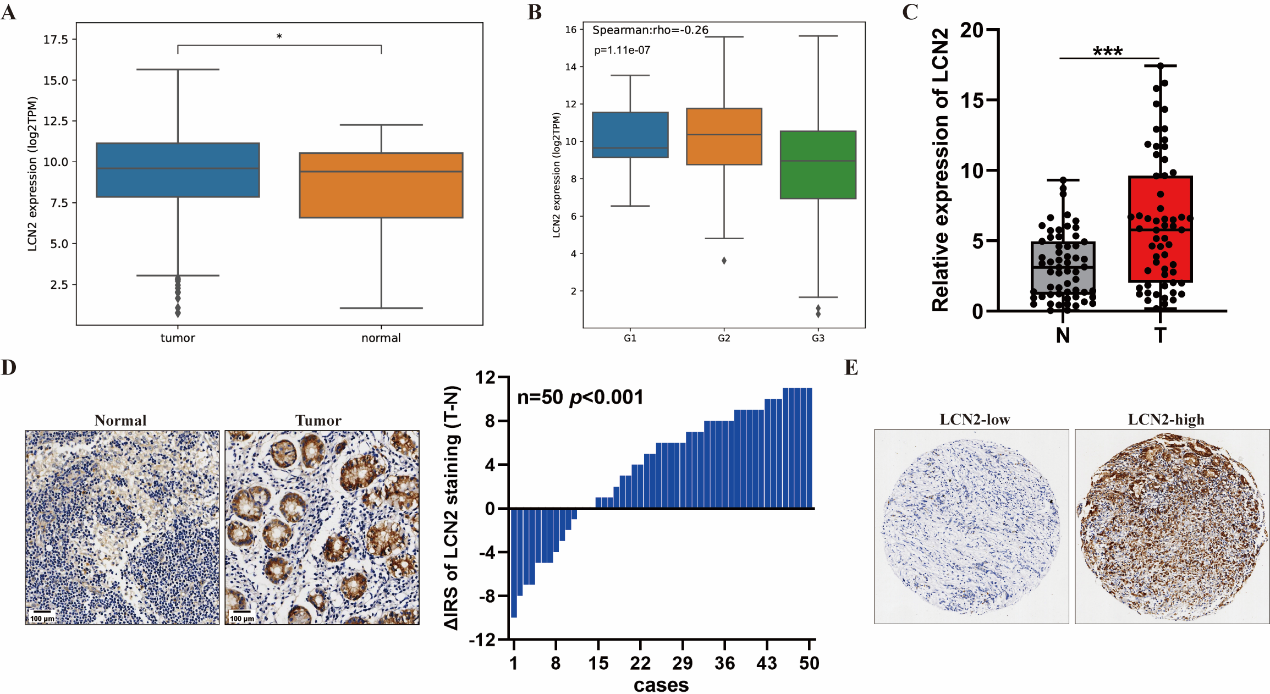


**Supplementary Fig. 1. LCN2 is aberrantly expressed in GC and correlates with the positive prognosis of patients**

(A) mRNA Expression levels of LCN2 in GC tissues and normal tissues of the TCGA database.

(B) TCGA data showed that LCN2 expression negatively correlated with GC grade.

(C) mRNA Expression levels of LCN2 in GC tissues and their paired adjacent normal tissues were detected by RT-qPCR (n=59, *p*<0.001).

(D) Representative images of IHC staining for LCN2 in GC tissues and their paired adjacent normal tissues and differential distribution of LCN2 immunoreactivity score (IRS) (ΔIRS=IRST-IRSN) (n=50, *p*<0.001).

(E) Representative images of IHC staining for LCN2 in tumor tissue microarray.

**p* < 0.05, ***p* < 0.01, ****p* < 0.001. Data are expressed as mean ± SD of three independent experiments.


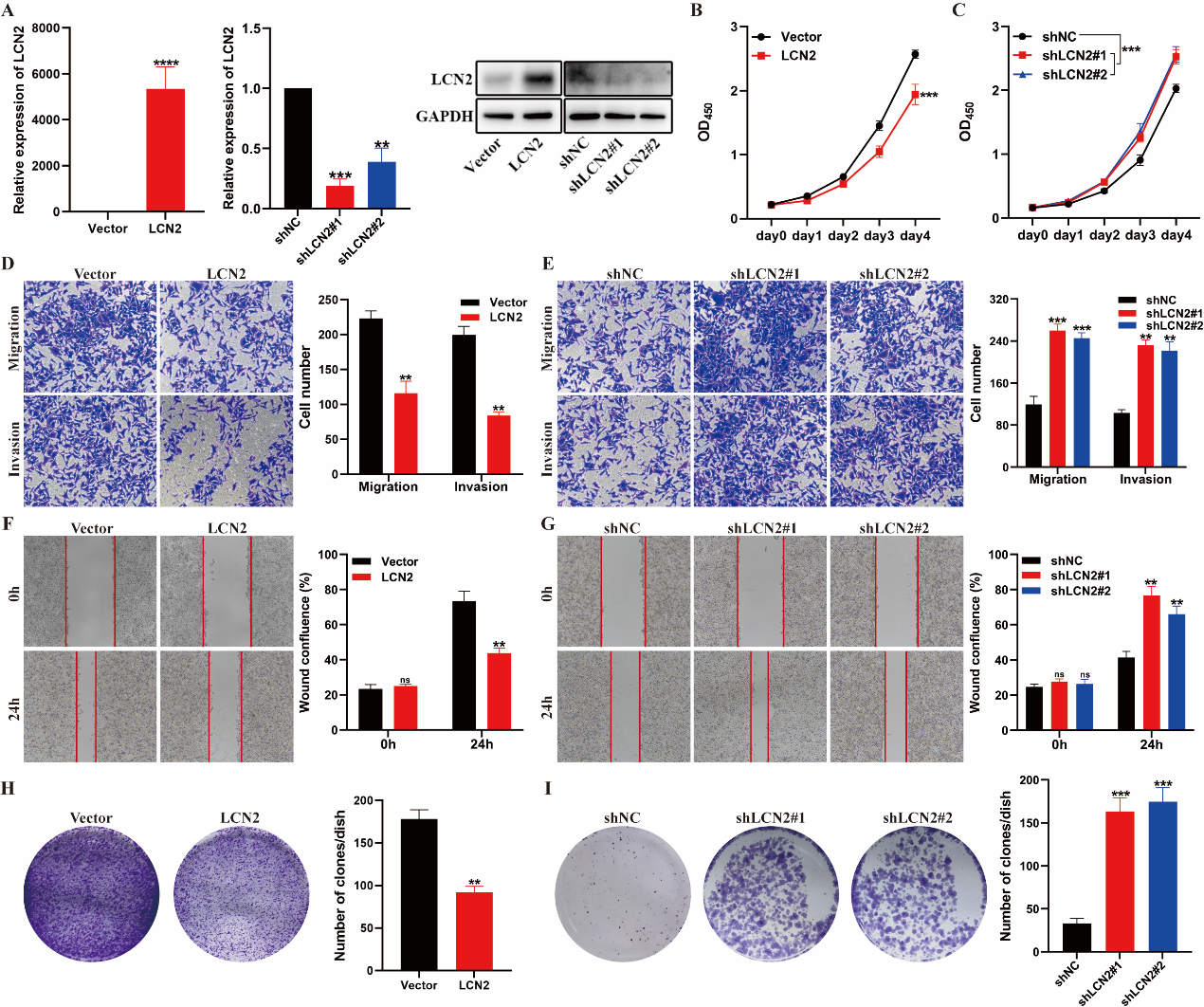


**Supplementary Fig. 2. LCN2 inhibits proliferation, migration, and invasion** **capabilities of MGC803 cell *in vitro***

(A) The overexpression and knockdown efficiencies of LCN2 in MGC803 cells, measured by RT-qPCR and western blotting.

(B, C) LCN2 overexpression suppressed MGC803 cells proliferation (B), while LCN2 knockdown promoted MGC803 cells proliferation (C) as assessed by CCK-8 assay.

(D, E) Representative images of transwell assay in transfected MGC803 cells (left panel) and quantitative results (right panel).

(F, G) Representative images of wound healing assay in transfected MGC803 cells (left panel) and quantitative results (right panel).

(H, I) Representative images of colony formation assay in transfected MGC803 cells (left panel) and quantitative results (right panel).

**p* < 0.05, ***p* < 0.01, ****p* < 0.001. Data are expressed as mean ± SD of three independent experiments.


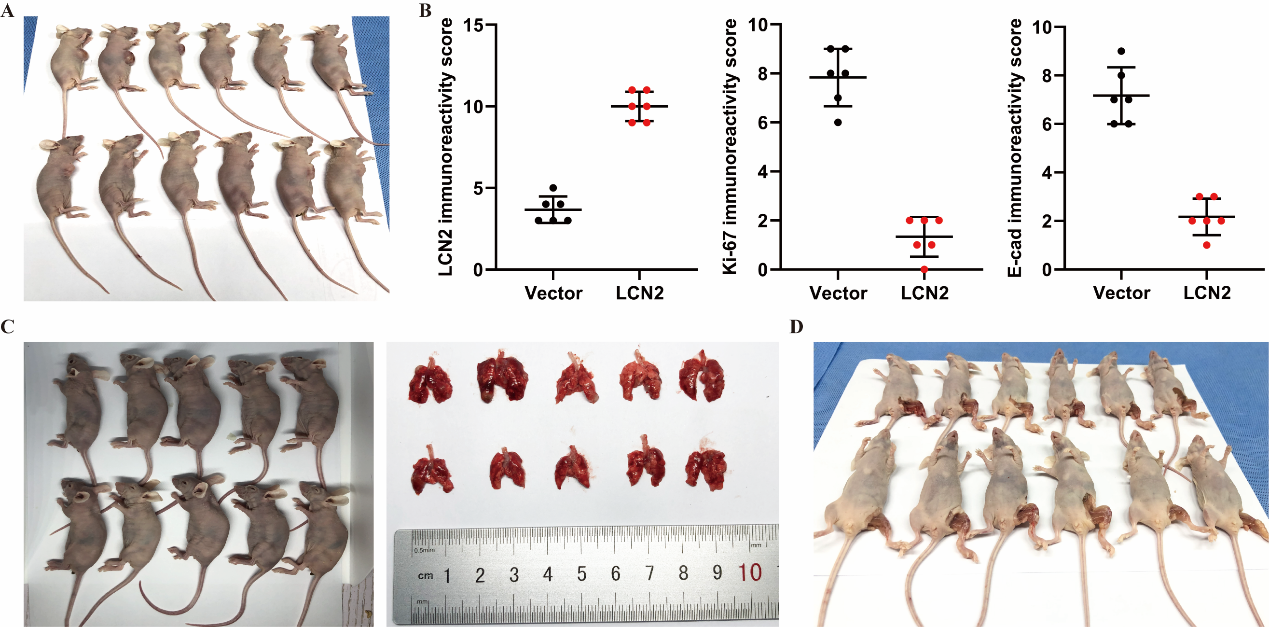


**Supplementary Fig. 3. The images of animal studies**

(A) The representative images of animals in xenograft studies in **Fig. 3A**.

(B) The quantitative results of IHC staining in **Fig. 3D**

(C) The representative images of animals and images of lung in xenograft studies in **Fig. 3E**.

(D) The representative images of animals in xenograft studies in **Fig. 3I**.


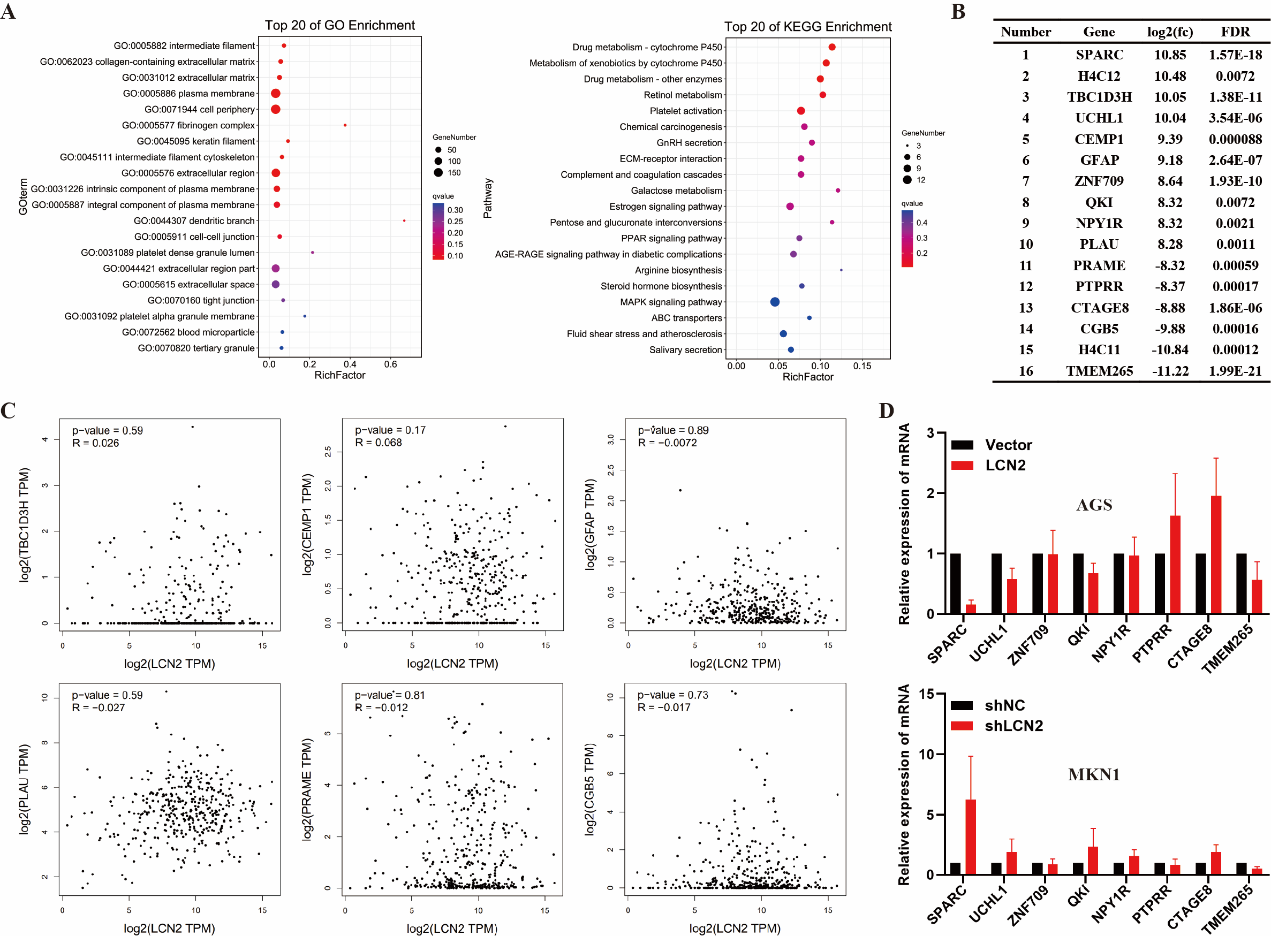


**Supplementary Fig. 4.** **SPARC is a pivotal downstream target of LCN2 in GC**

(A) GO enrichment analysis and KEGG pathway enrichment on different expression genes of LCN2 knock-downed MKN1 cells.

(B) The 16 genes change greater than 8 and *p-*values less than 0.01 of the RNA-seq between negative control cells and LCN2 knockdown cells.

(C) the correlation between LCN2 and these genes above in STAD of the GEPIA database.

(D) RT-qPCR was used to measure the mRNA levels of these genes above in LCN2 overexpressed cells, LCN2 knockdown cells, and corresponding control cells.


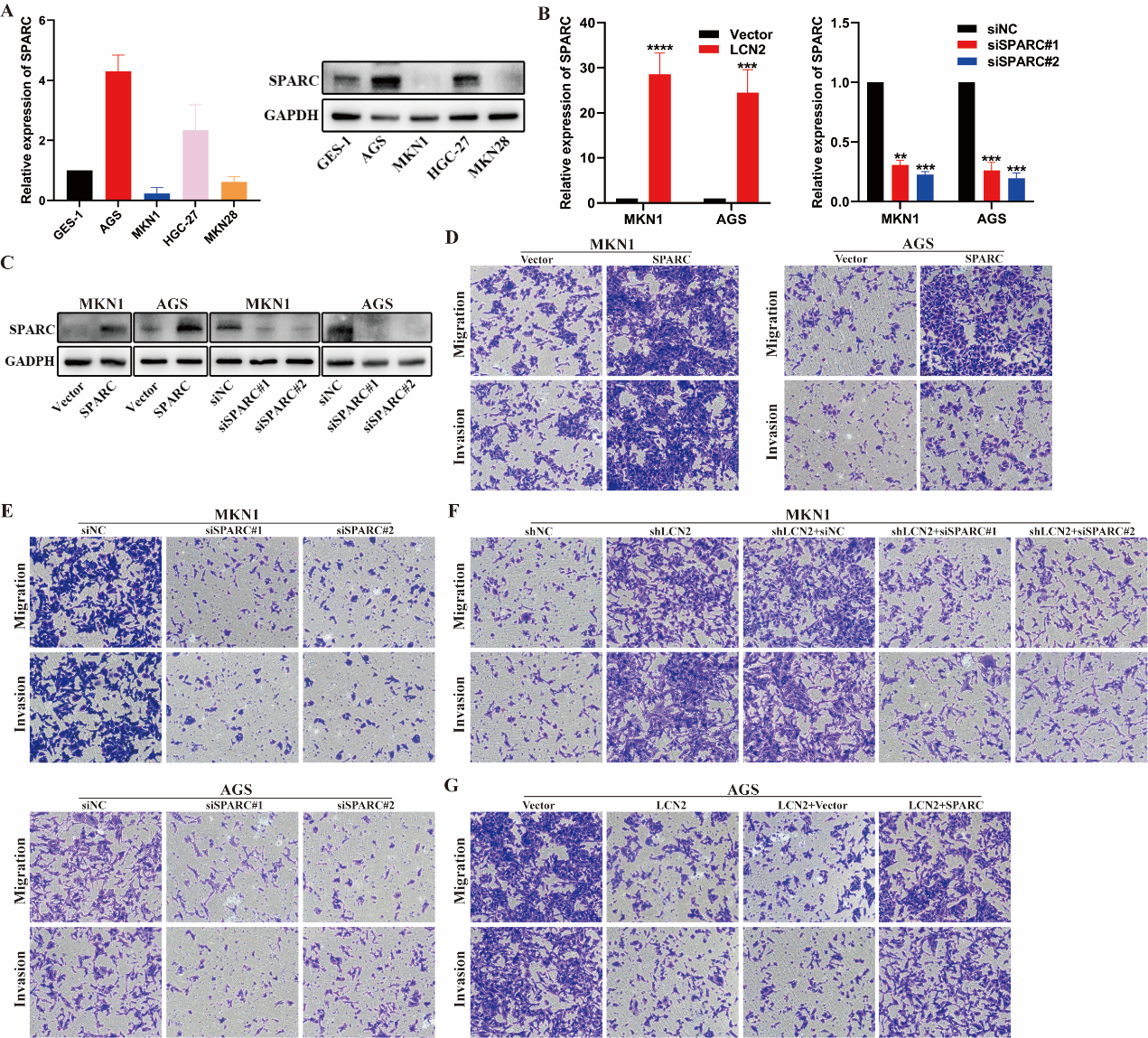


**Supplementary Fig. 5. SPARC is a pivotal downstream target of LCN2 in GC**

(A) The mRNA and protein expression levels of SPARC in GES-1 and different GC cell lines.

(B, C) The overexpression and knockdown efficiencies of SPARC in MKN1 and AGS cells, measured by RT-qPCR (B) and western blotting (C).

(D) Representative images of transwell assay in SPARC overexpressed MKN1 and AGS cells.

(E) Representative images of transwell assay in SPARC knockdown MKN1 and AGS cells.

(F) Representative images of transwell assay in LCN2 knockdown cells with SPARC knockdown.

(G) Representative images of transwell assay in LCN2 overexpressed cells with SPARC overexpressed.

**p* < 0.05, ***p* < 0.01, ****p* < 0.001. Data are expressed as mean ± SD of three independent experiments.


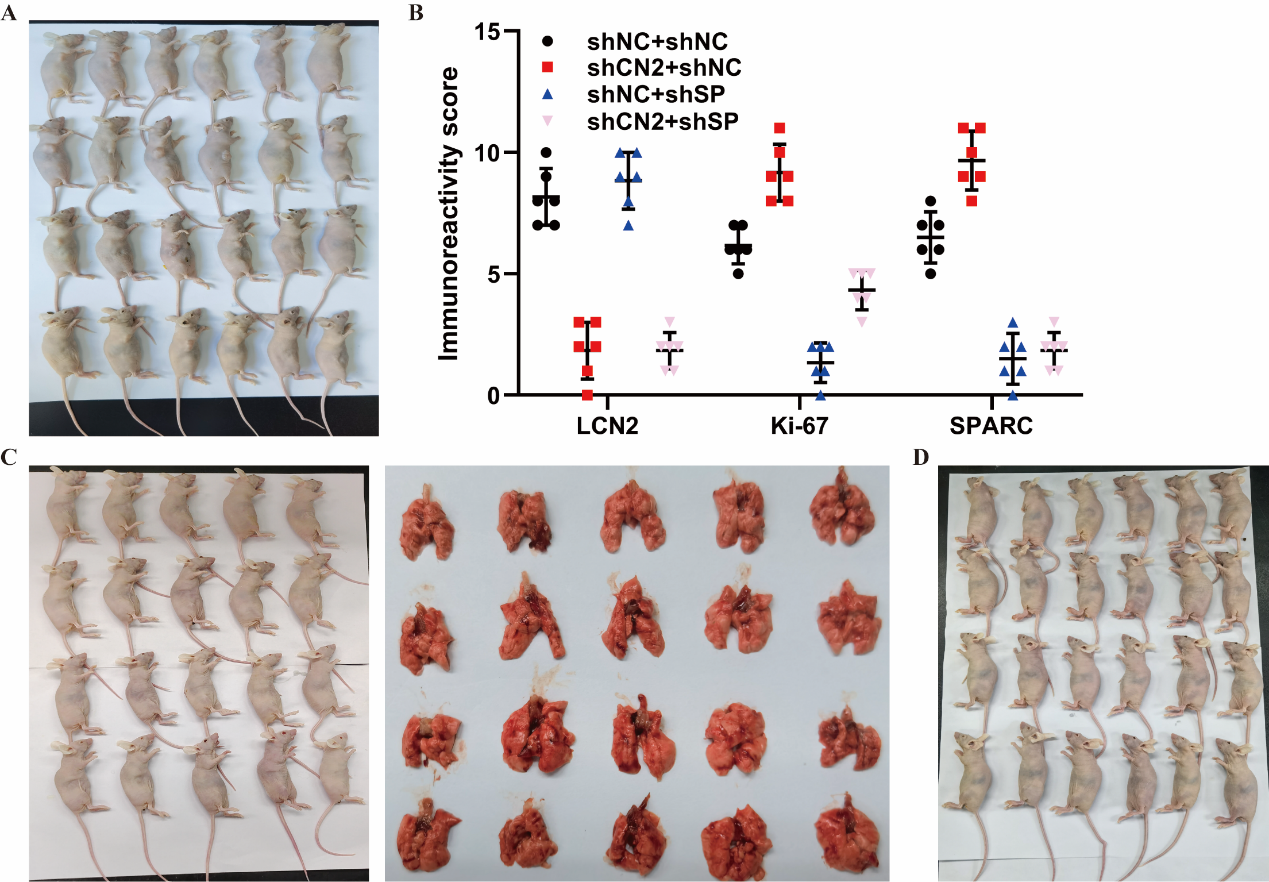


**Supplementary Fig. 6. The images of animal studies**

(A) The representative images of animals in xenograft studies in **Fig. 5A**.

(B) The quantitative results of IHC staining in **Fig. 5D**

(C) The representative images of animals and images of lung in xenograft studies in **Fig. 5E**.

(D) The representative images of animals in xenograft studies in **Fig. 5G**.


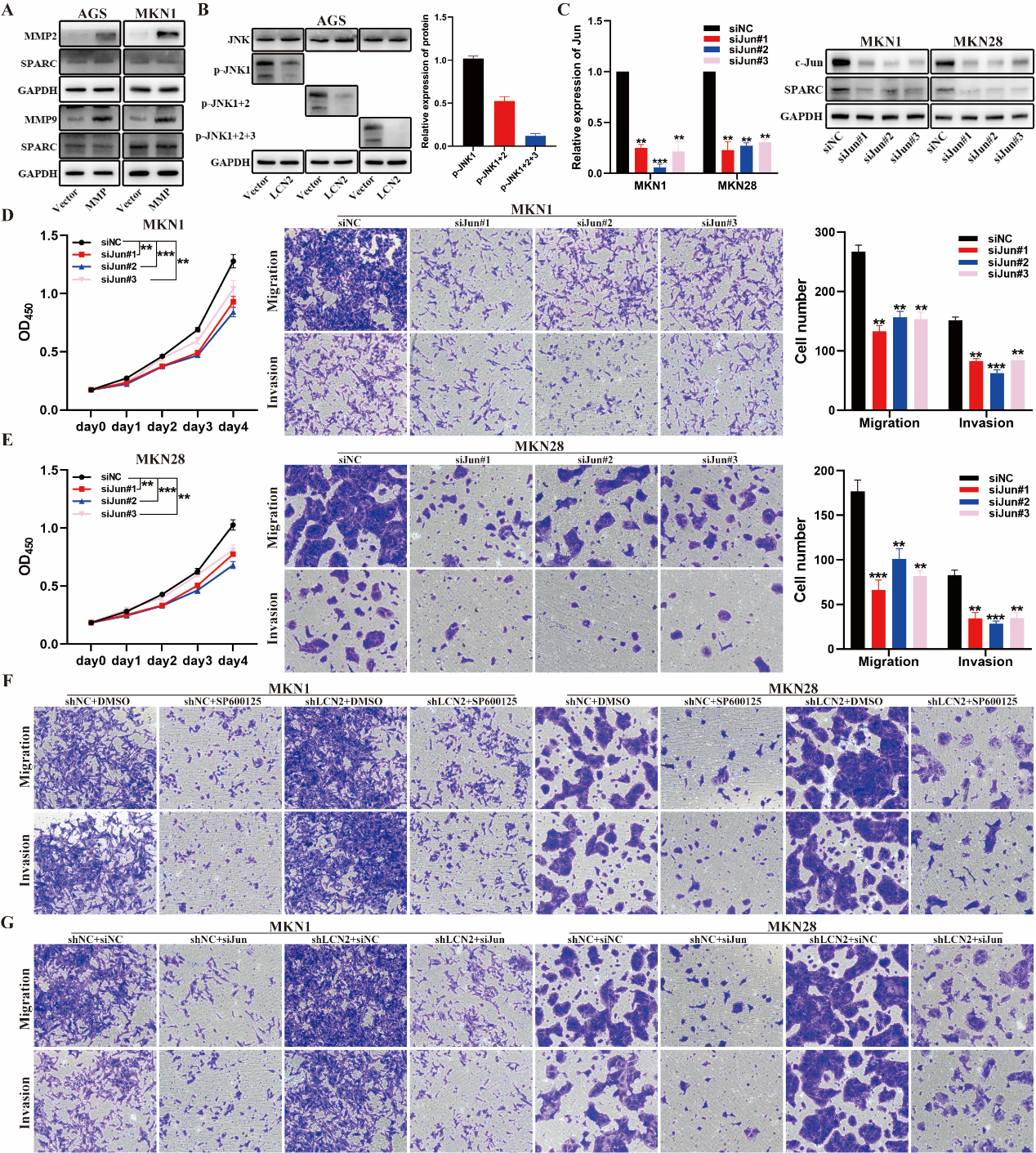


**Supplementary Fig. 7. LCN2** **downregulates SPARC expression by inhibiting the JNK/c-Jun signaling pathway**

(A) Detection of SPARC protein levels in GC cells with MMP2 or MMP9 overexpression in AGS cells using western blot.

(B) Detection of p-JNK1, p-JNK1+2, and p-JNK1+2+3 protein levels in GC cells with MMP2 or MMP9 overexpression in AGS cells using western blot.

(C) The knockdown efficiencies of c-Jun in MKN1 and MKN28 cells, measured by RT-qPCR and western blotting.

(D) Knockdown of c-Jun inhibited the proliferation and metastasis ability of MKN1 cells as assessed by CCK-8 and transwell assay.

(E) Knockdown of c-Jun inhibited the proliferation and metastasis ability of MKN28 cells as assessed by CCK-8 and transwell assay.

(F) Representative images of migration and invasion cells on LCN2 knockdown cells with SP600125 treatment in transwell assays.

(G) Representative images of migration and invasion cells on LCN2 knockdown cells with c-Jun knockdown in transwell assays.

**p* < 0.05, ***p* < 0.01, ****p* < 0.001. Data are expressed as mean ± SD of three independent experiments.


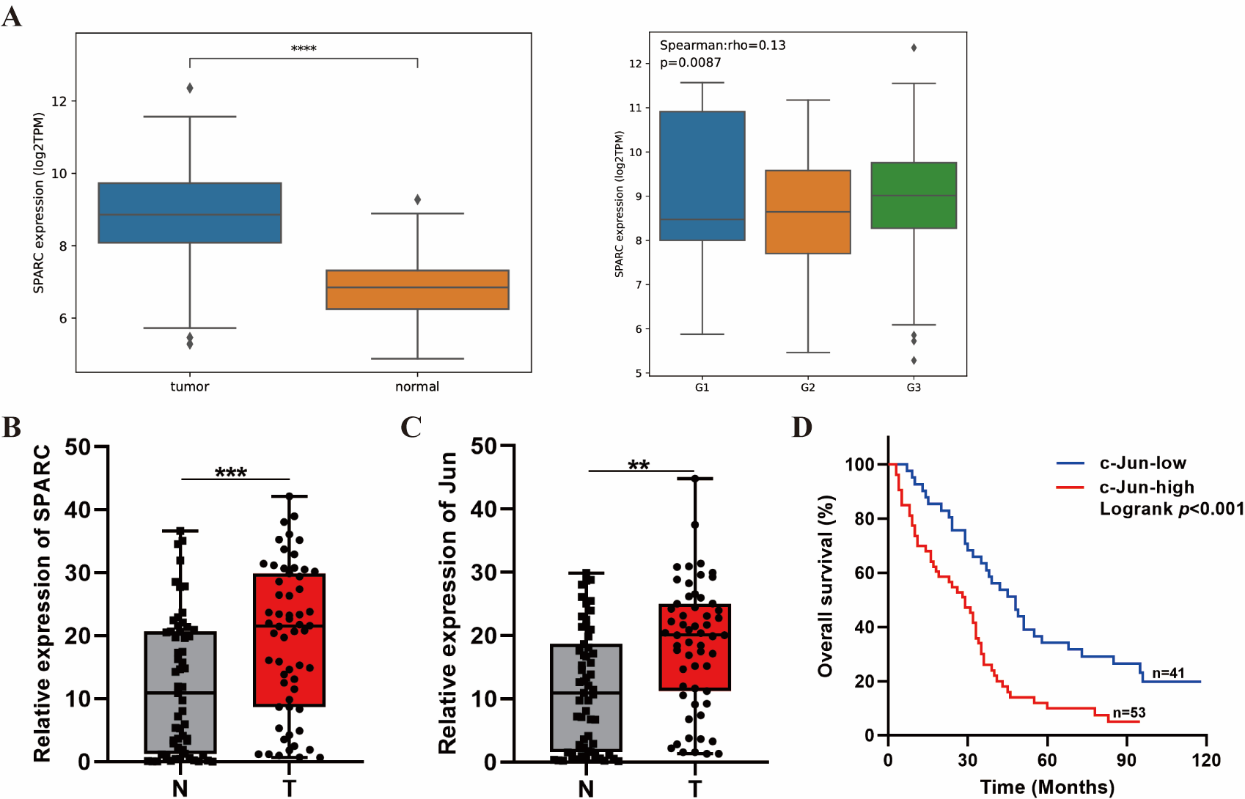


**Supplementary Fig. 8. Clinical relevance of LCN2, SPARC and c-Jun in GC patients.**

(A) TCGA data showing high expression of SPARC in GC tissues and positive correlation between SPARC overexpression and higher grade of GC.

(B) Expression levels of c-Jun in GC tissues and their paired adjacent normal tissues were detected by RT-qPCR (n=59, *p*<0.001).

(C) Expression levels of c-Jun in GC tissues and their paired adjacent normal tissues were detected by RT-qPCR (n=59, *p*<0.01).

(D) Kaplan–Meier survival analysis revealed that high expression of c-Jun was correlated with shorter OS times in GC patients (n=94, *p*<0.001; log-rank test).
