# Supplementary material for: Tumor-secreted LCN2 impairs gastric cancer progression via autocrine inhibition of the 24p3R/JNK/c-Jun/SPARC axis
Source: Cell Death Dis. 2024 Oct 18;15(10):756. doi: 10.1038/s41419-024-07153-z (PMC11489581; doi:10.1038/s41419-024-07153-z)
Supplement: Supplementary file 3 — Original western blots [file 41419_2024_7153_MOESM3_ESM.pdf]

Figure 1

Figure 1C

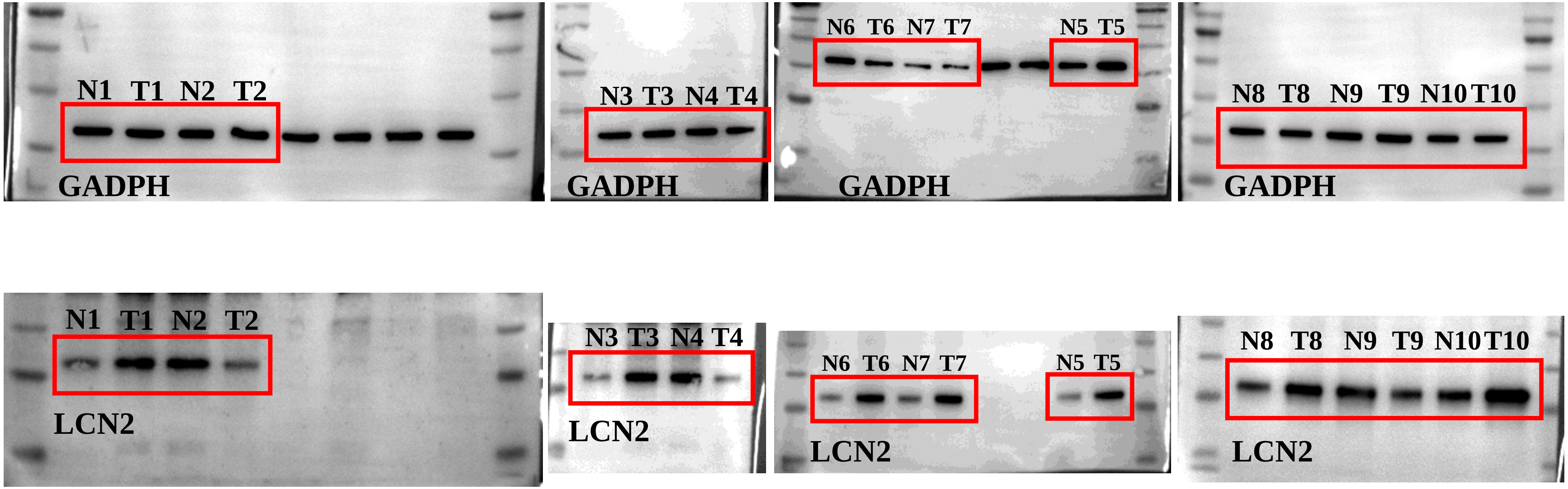

Figure 1F

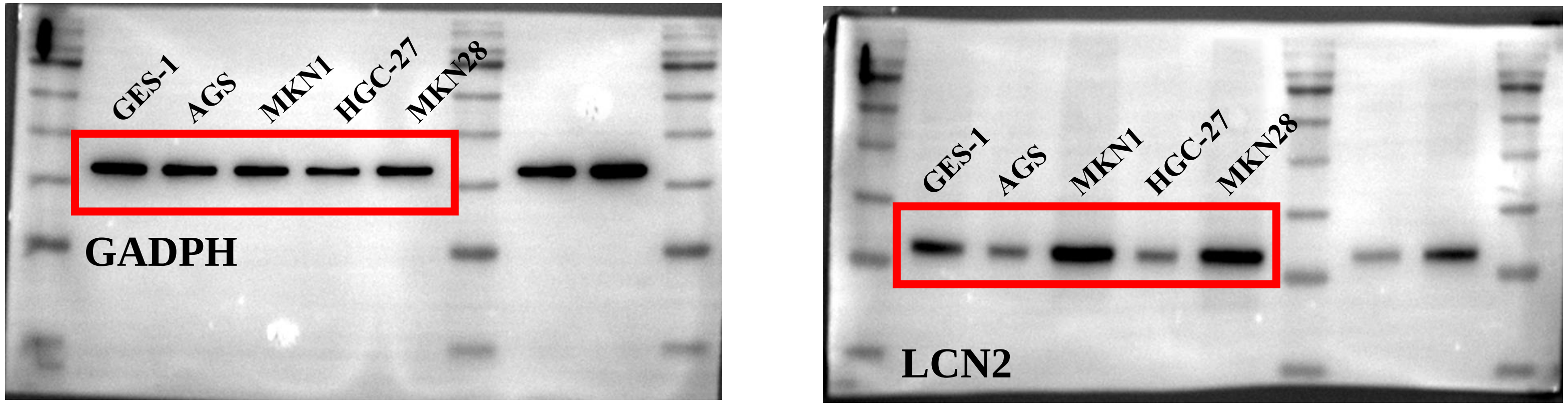

Figure 2

Figure 2A-B

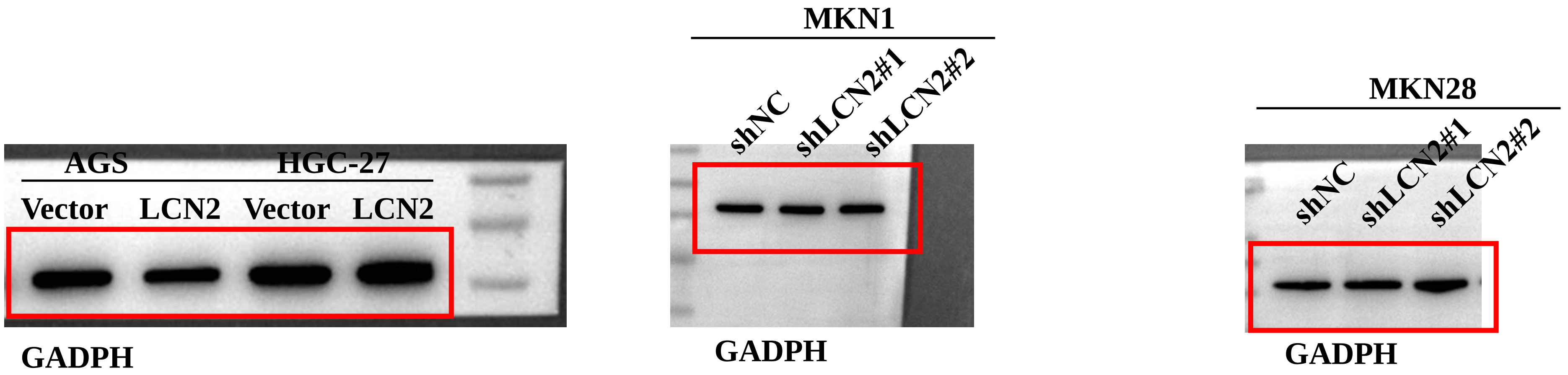

Figure 2A-B

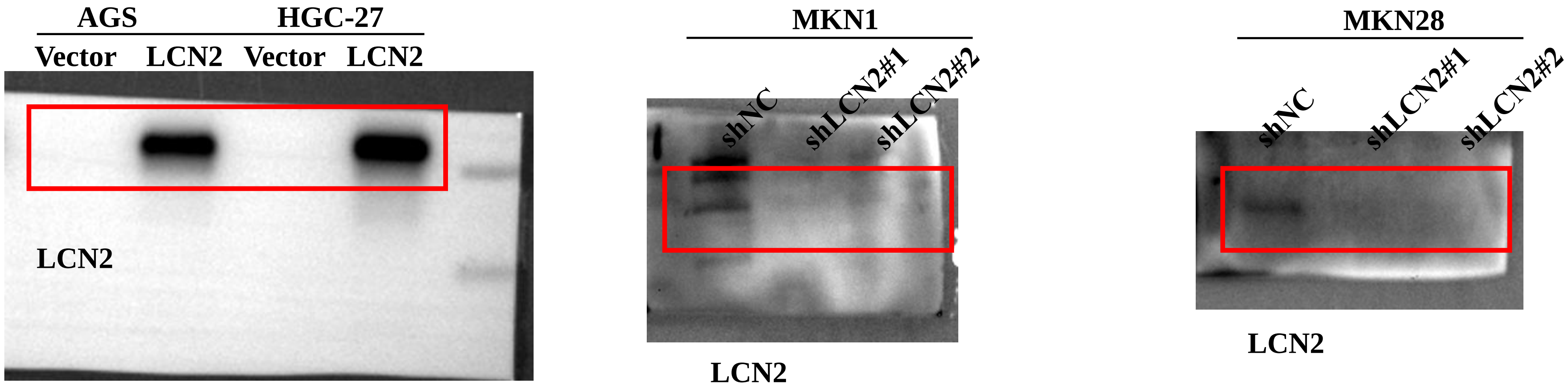

Figure 4

Figure 4C

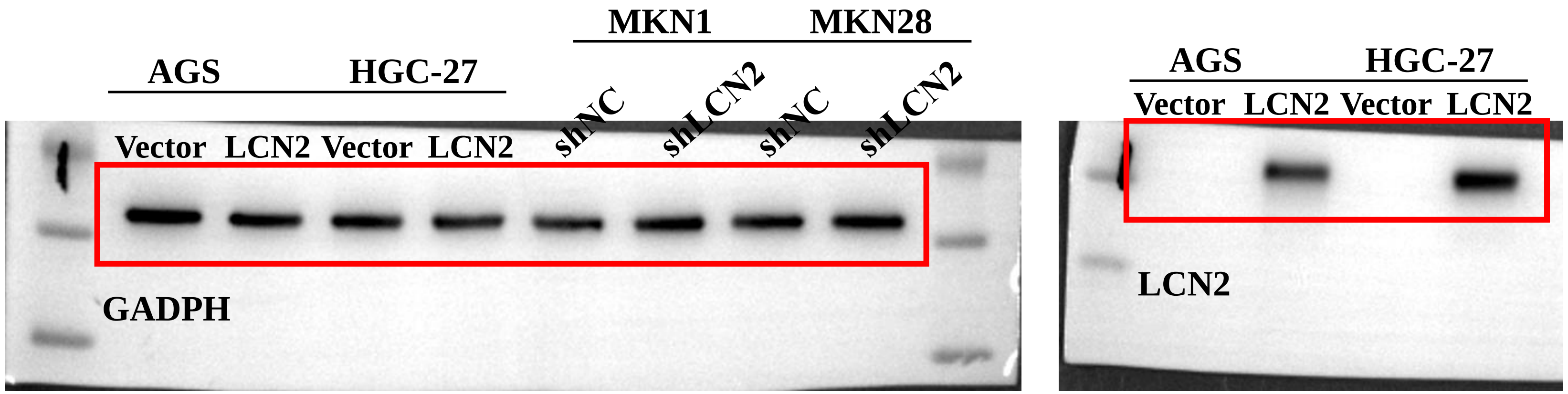

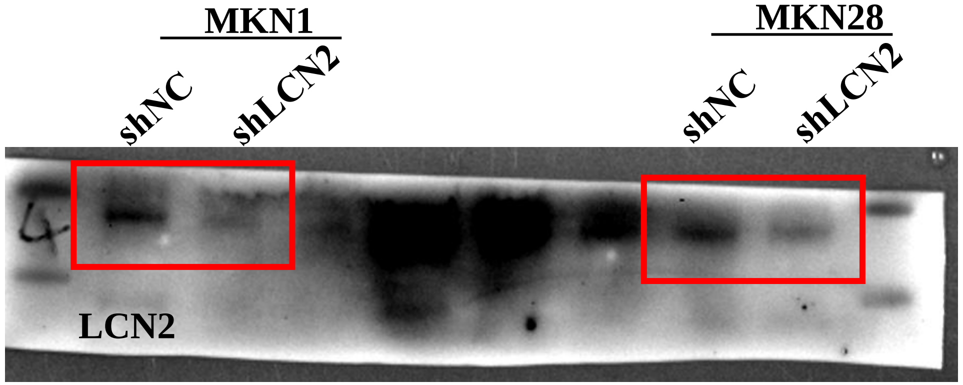

Figure 4C

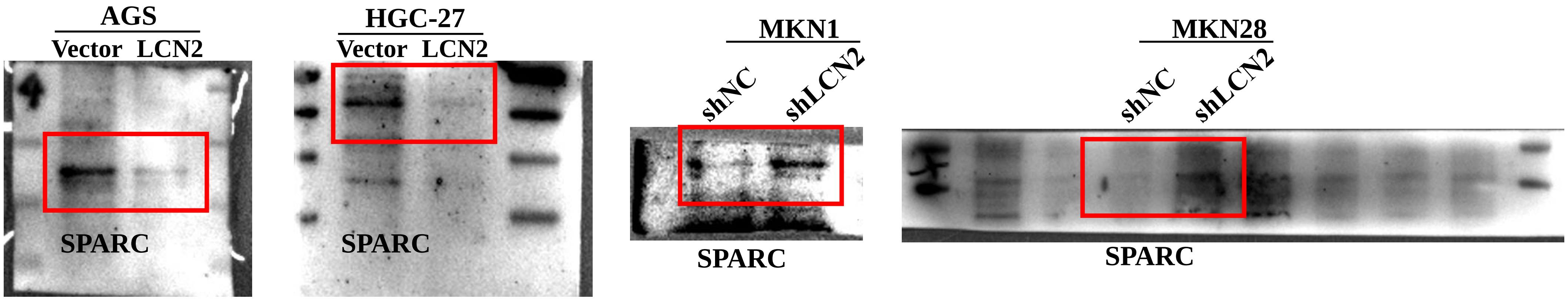

Figure 4

Figure 4I

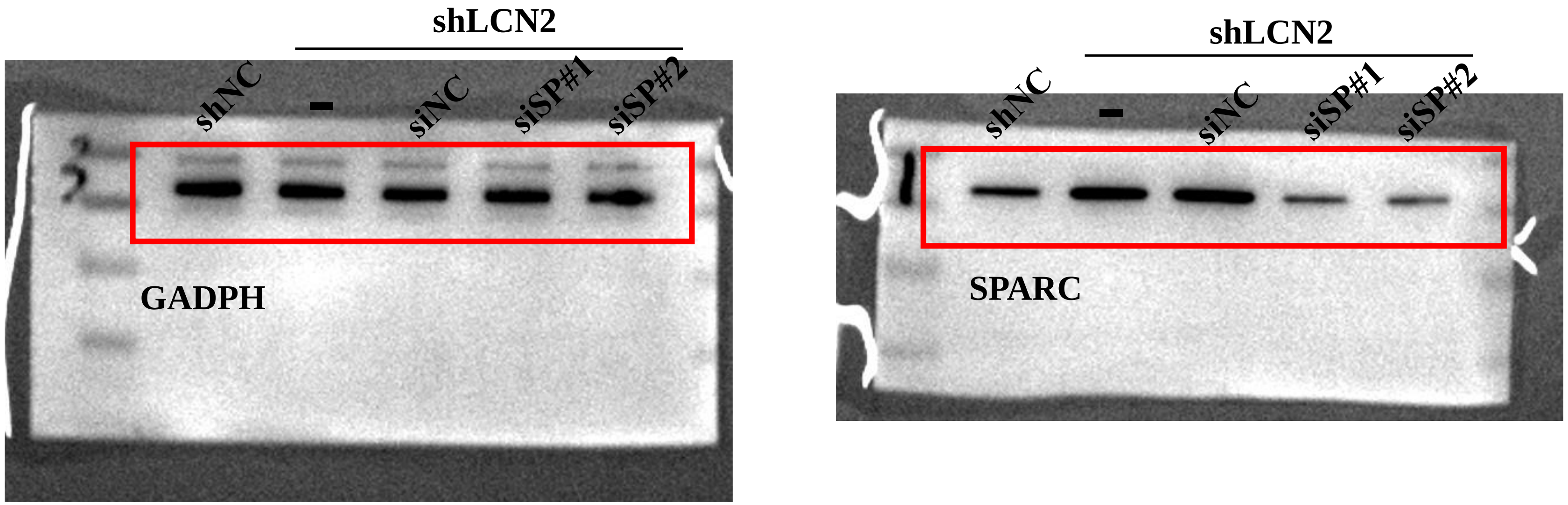

Figure 4K

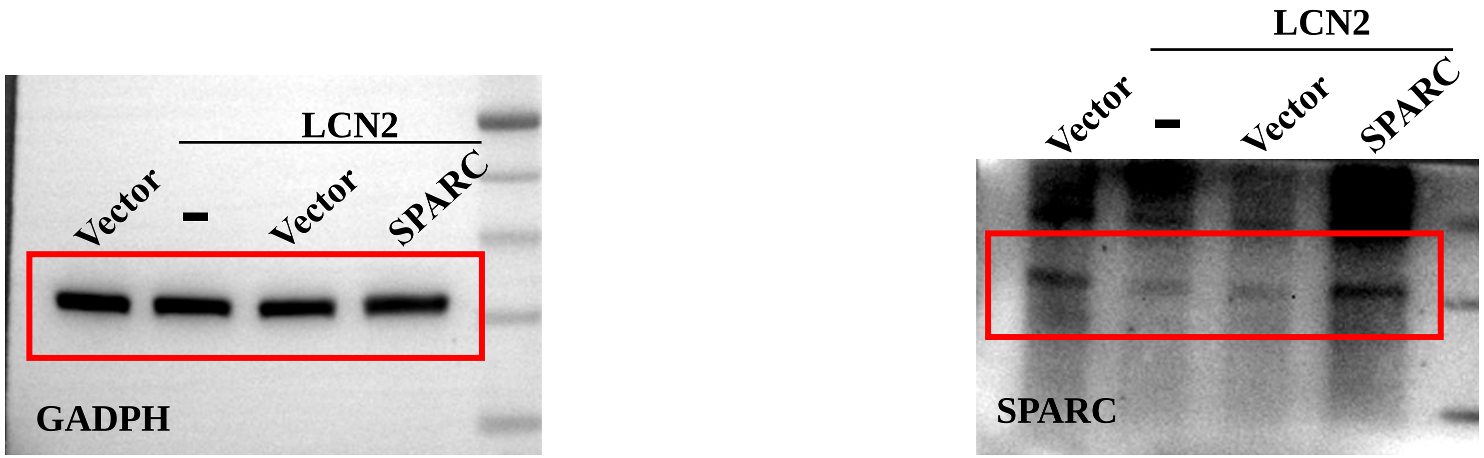

Figure 6

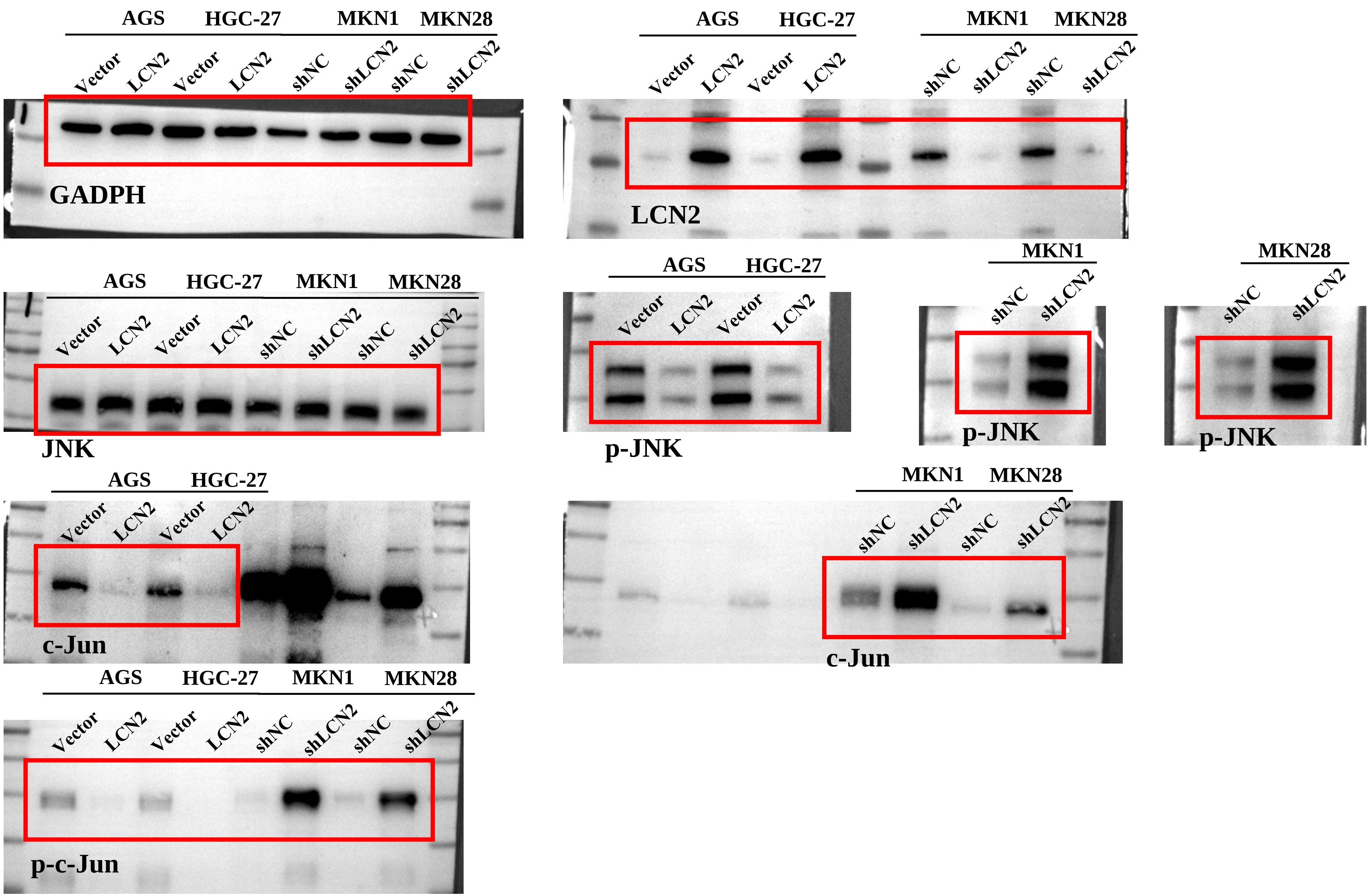

Figure 6B

Figure 6

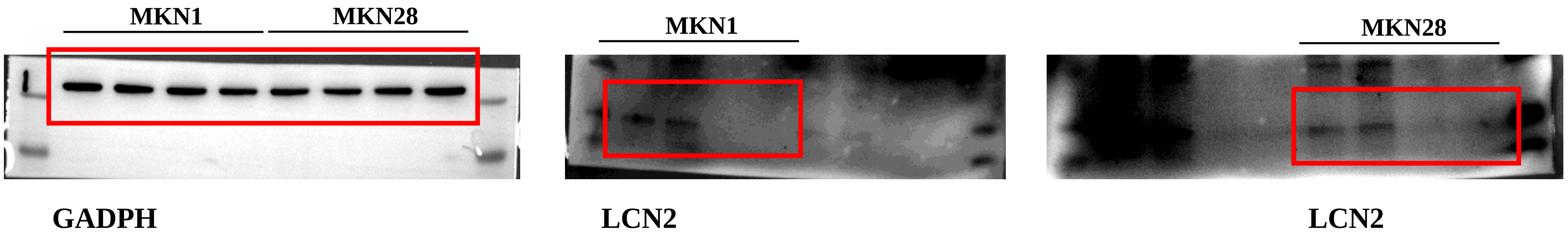

Figure 6C&E

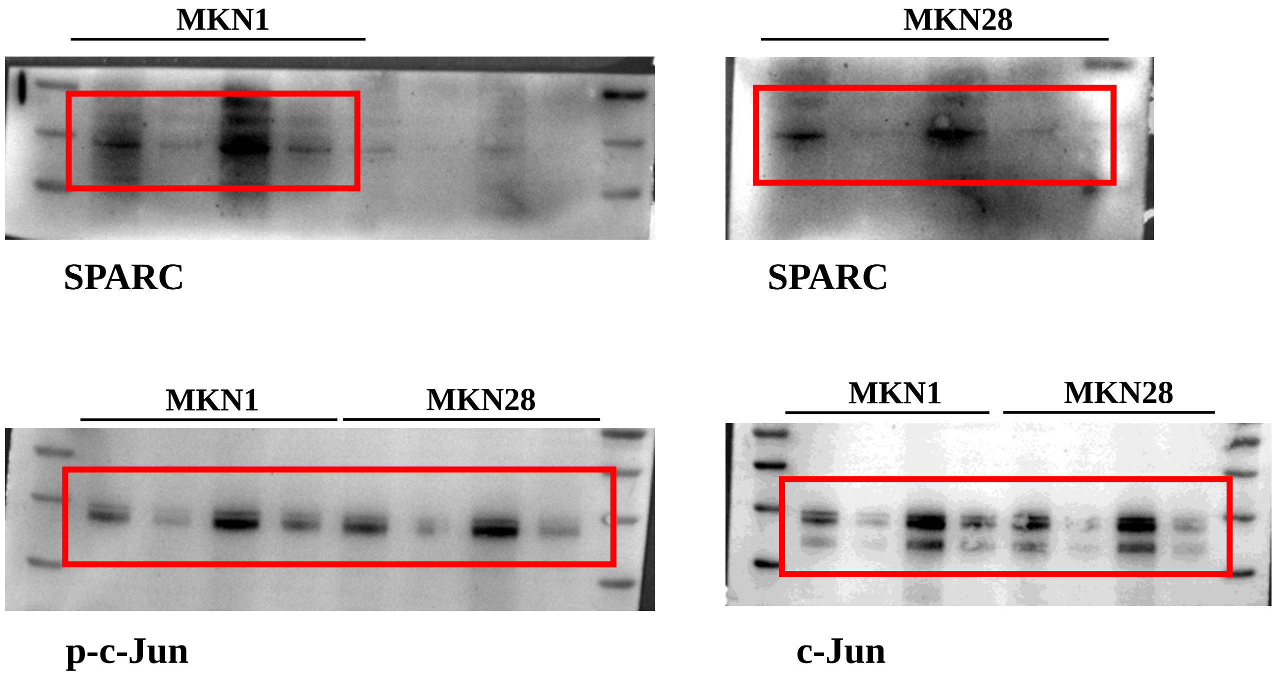

Figure 6

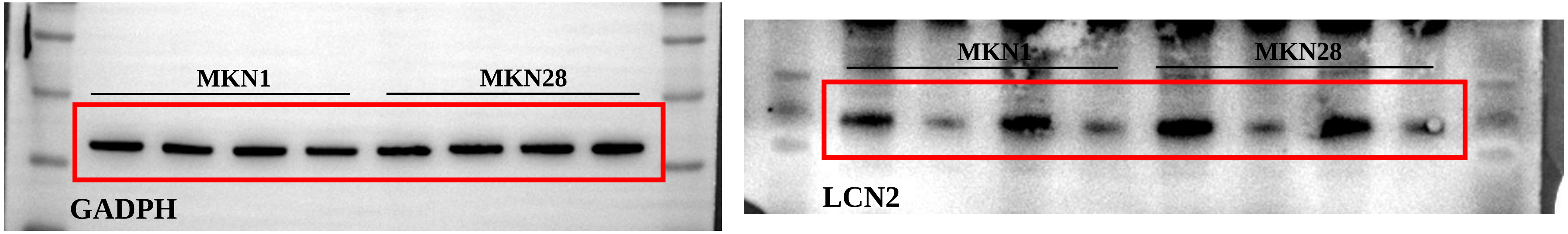

Figure 6G&I

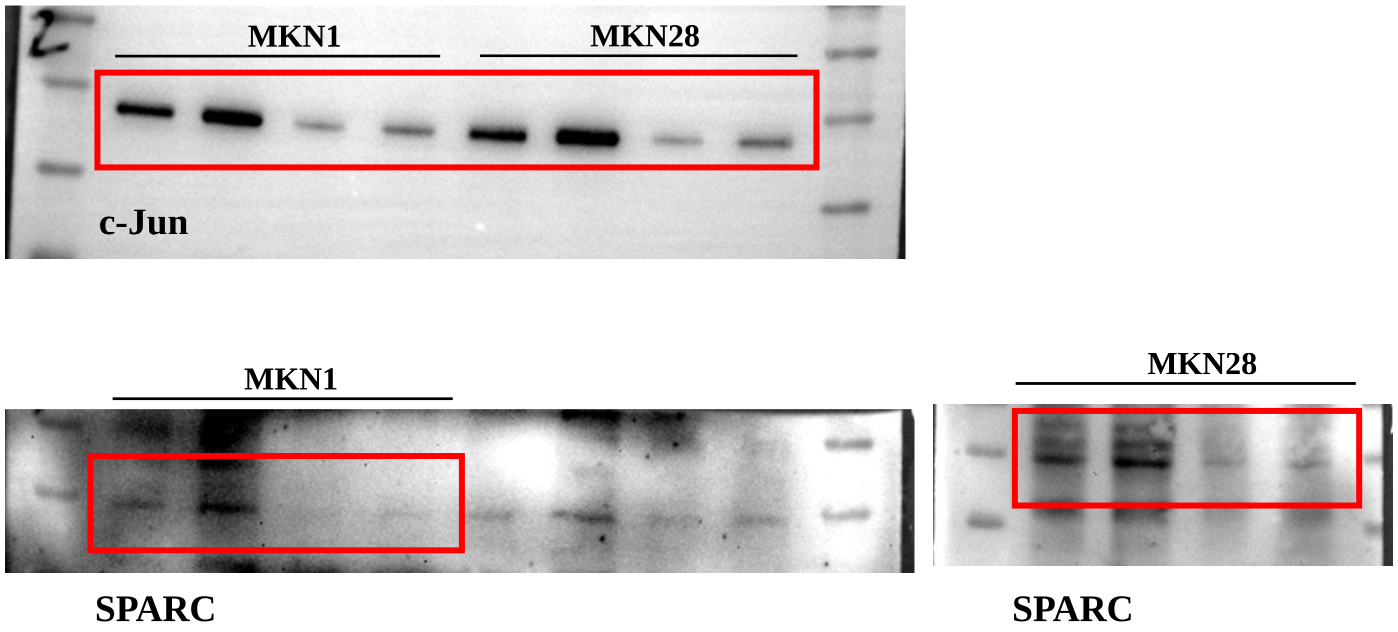

Figure 7

Figure 7A

AGS

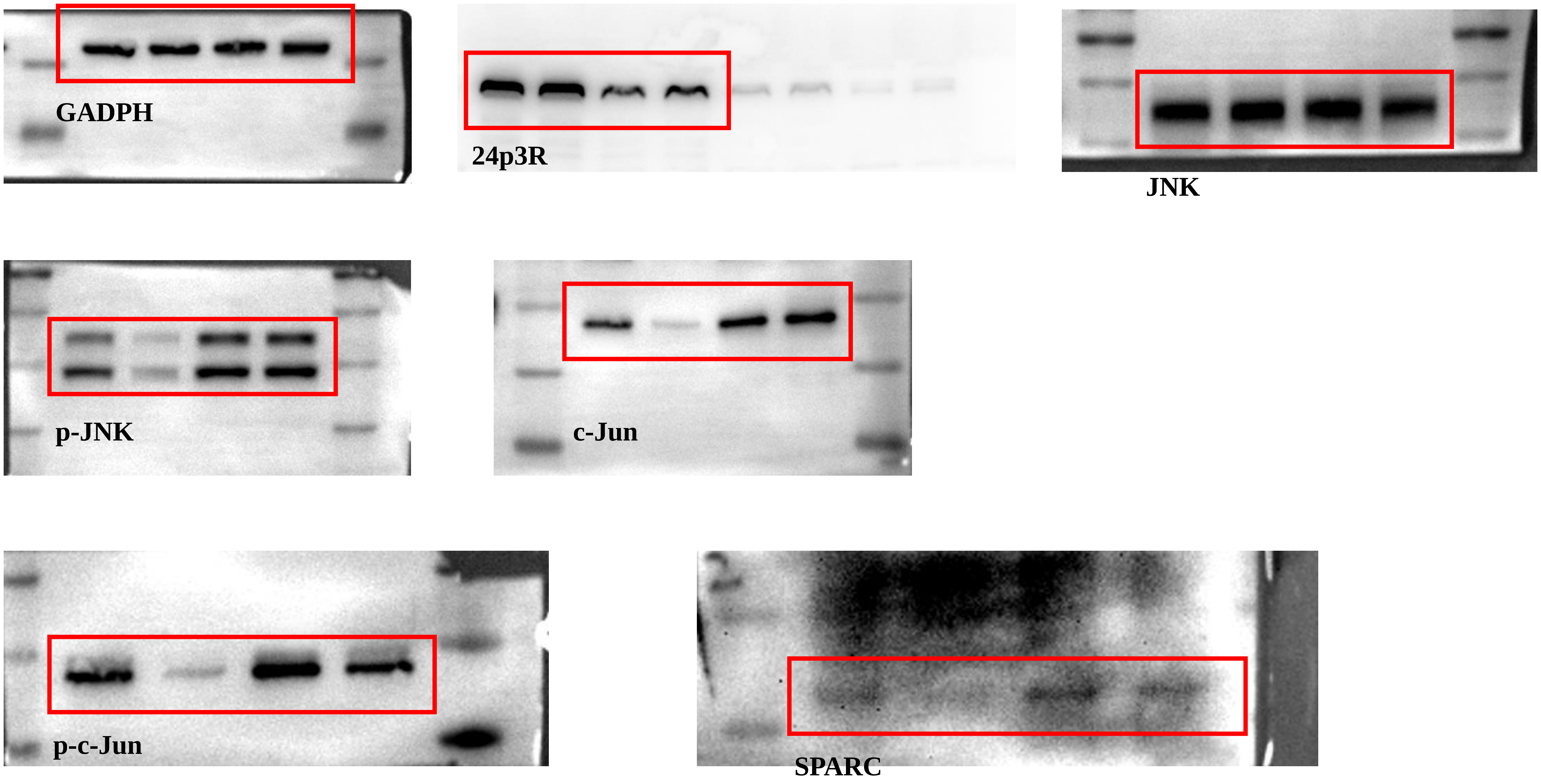

Figure 7

Figure 7A

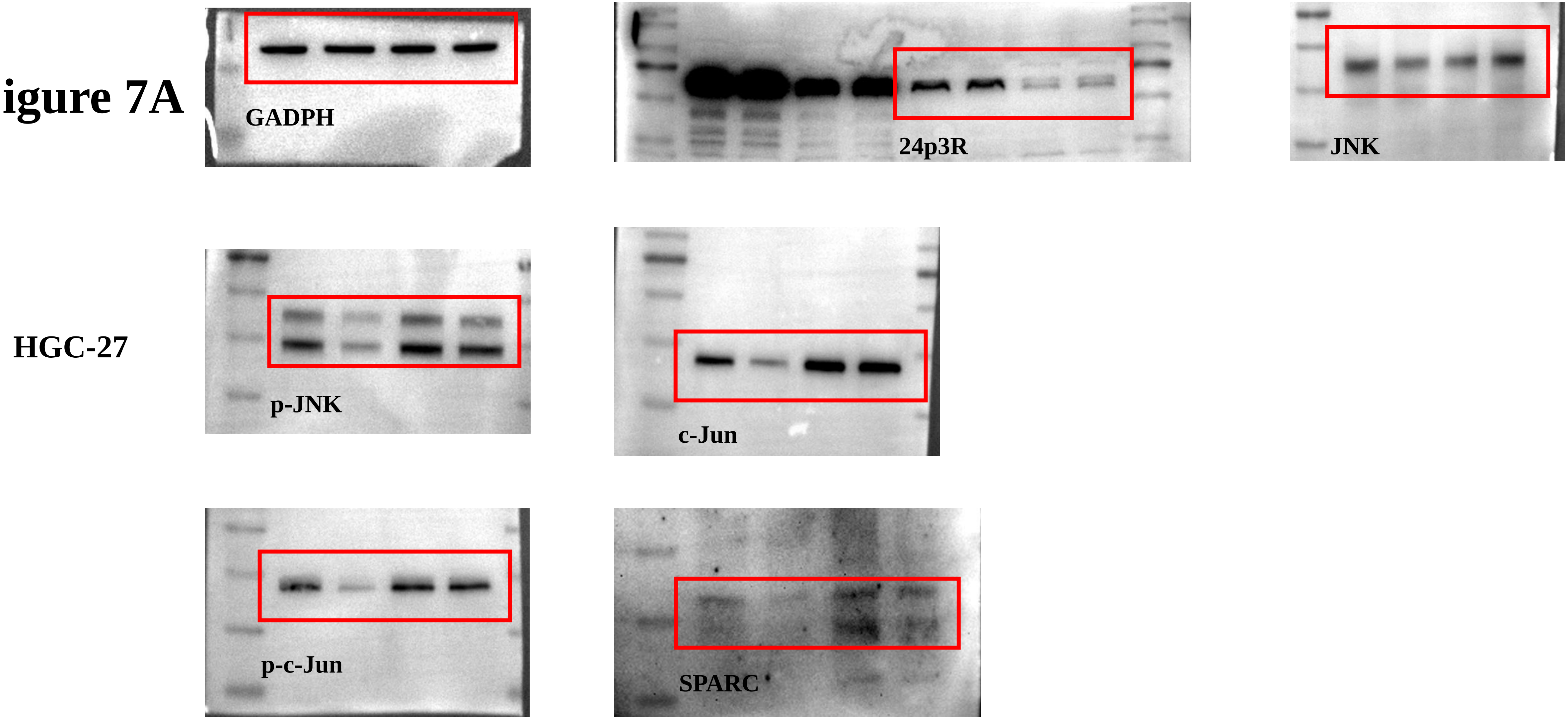

Figure 7

Figure 7B

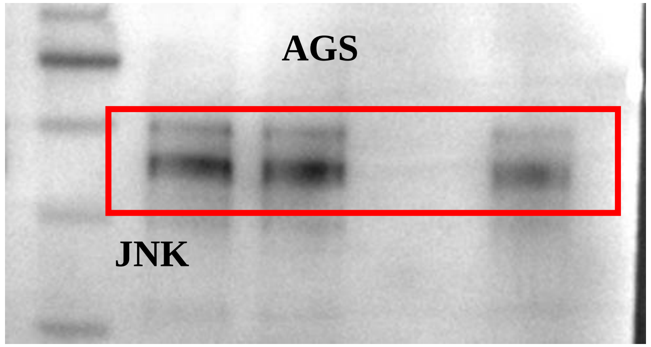

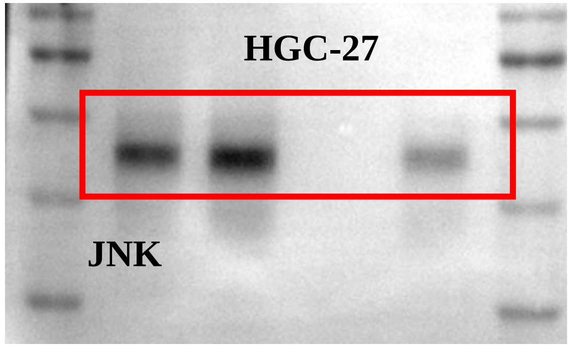

Figure 7C

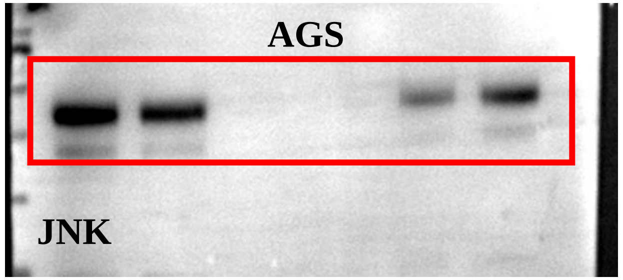

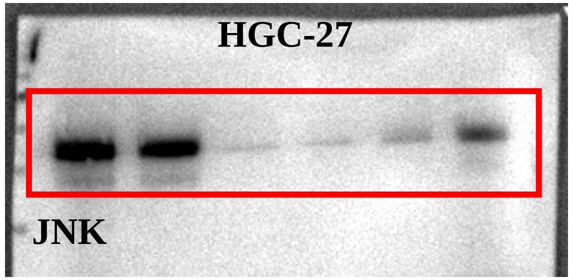

Figure 7D

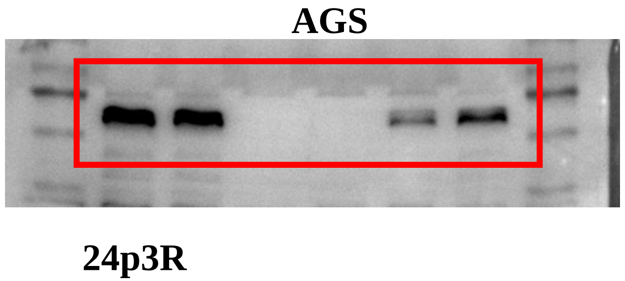

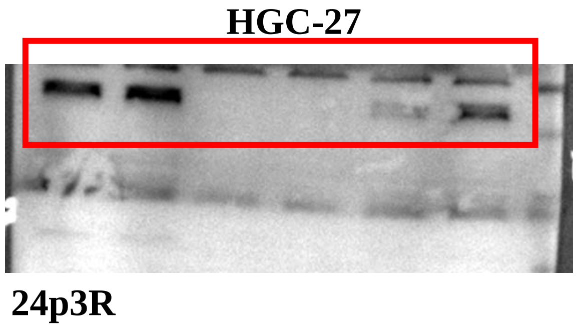

Figure 7

Figure 7F

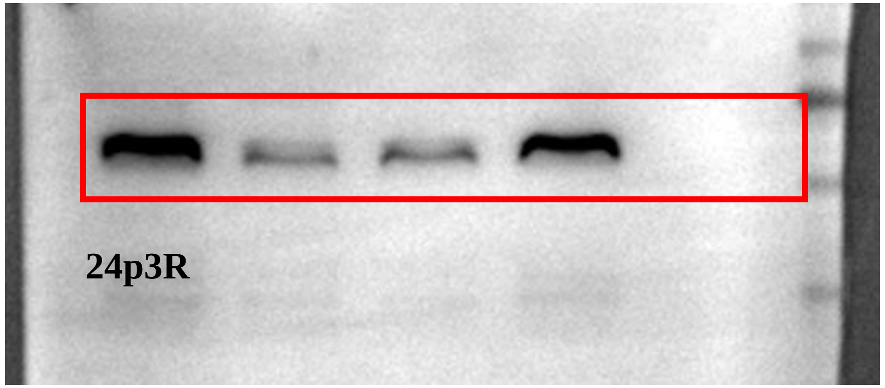

Figure 7G

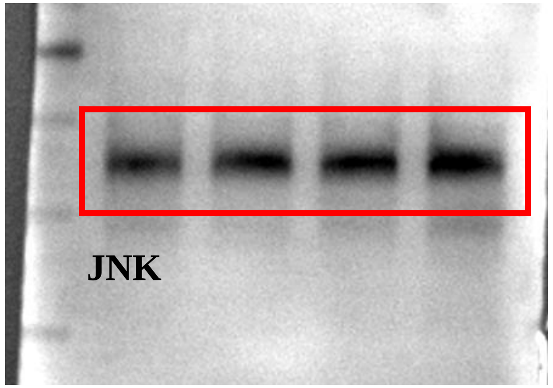

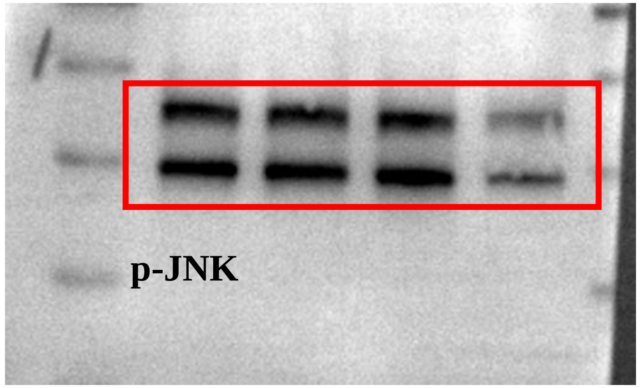

Figure 8

Figure 8B

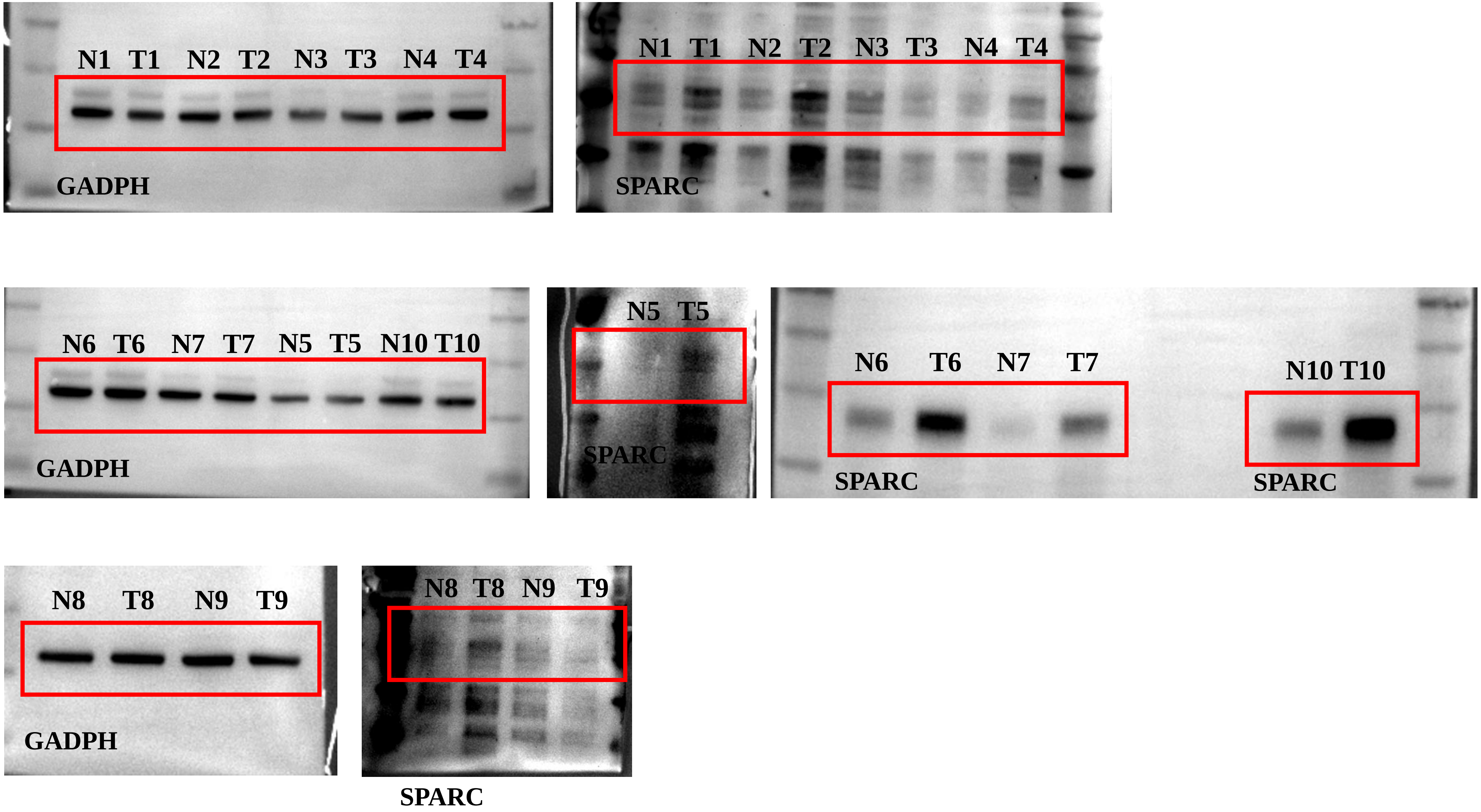

Figure S2

Figure S2A

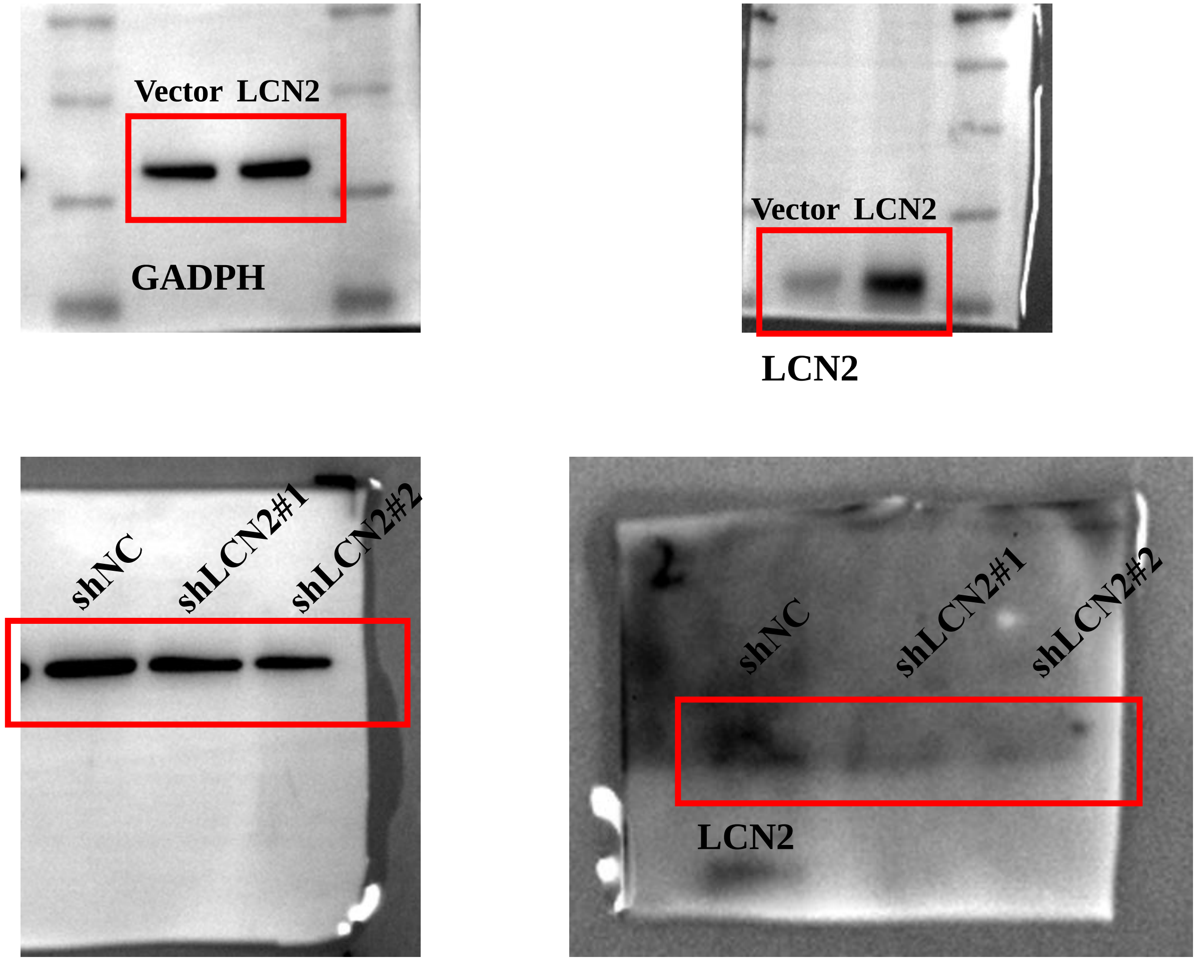

Figure S5

Figure S5A

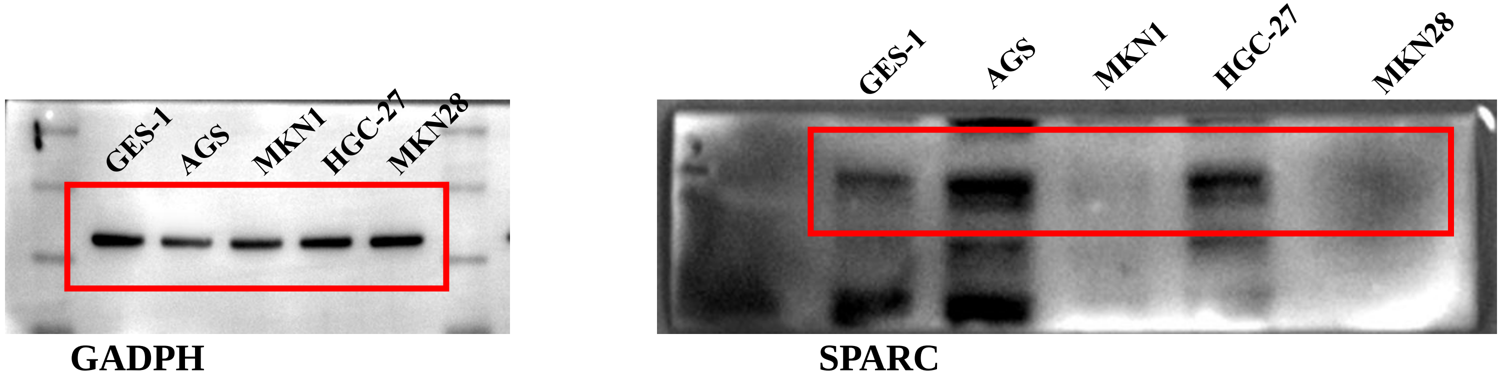

Figure S5B

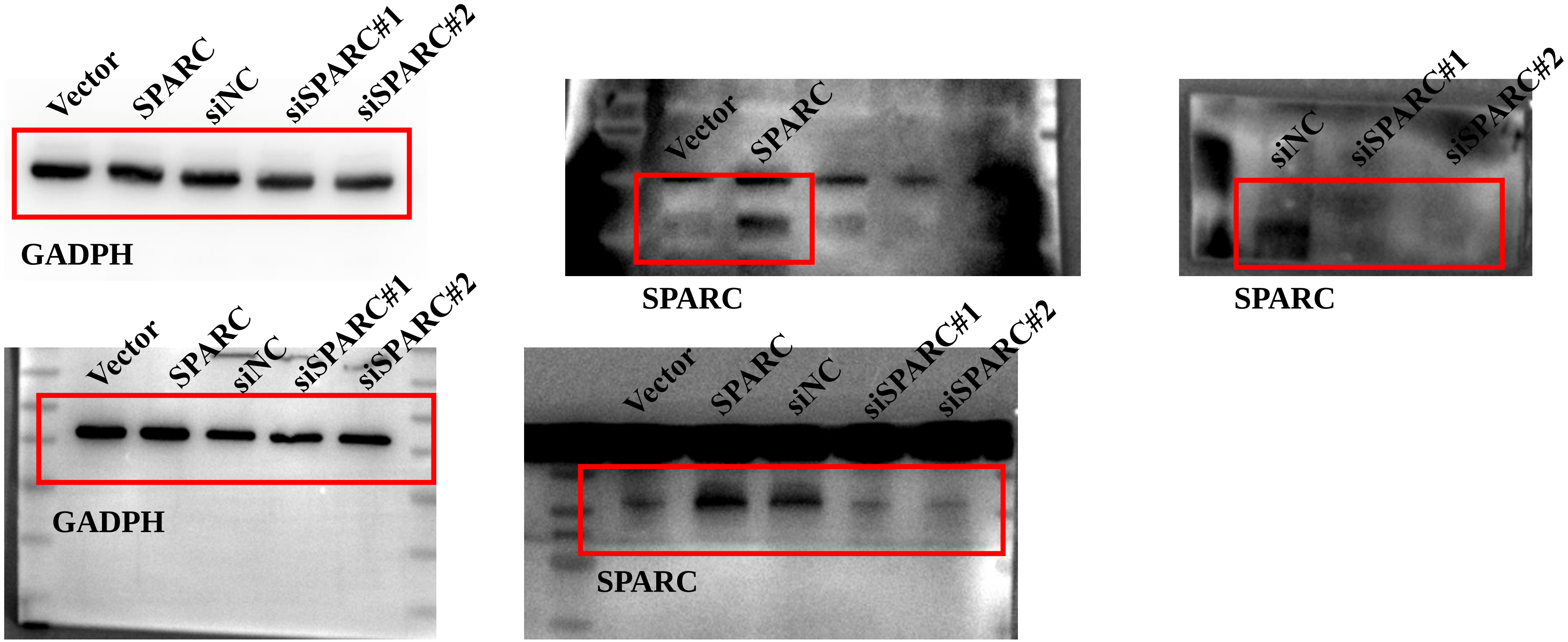

Figure S7

Figure S7A

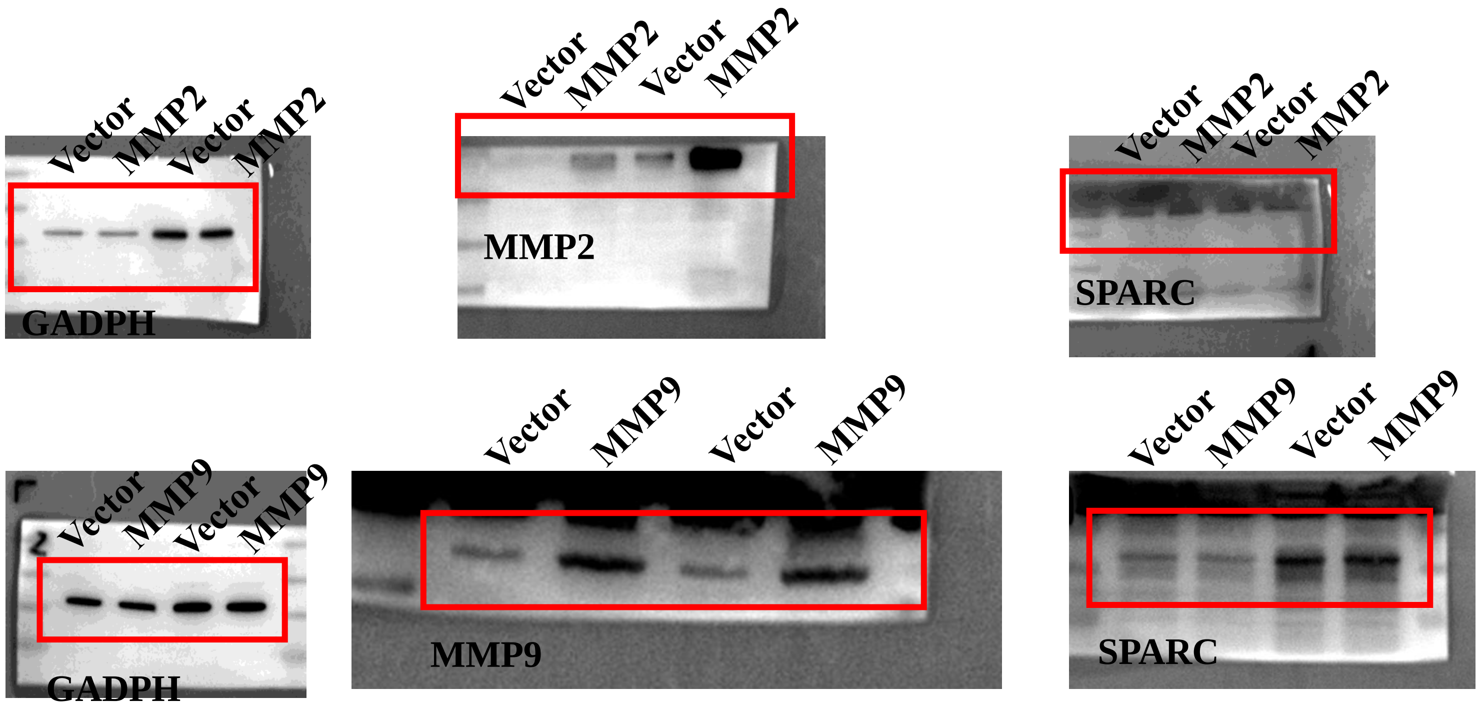

Figure S7B

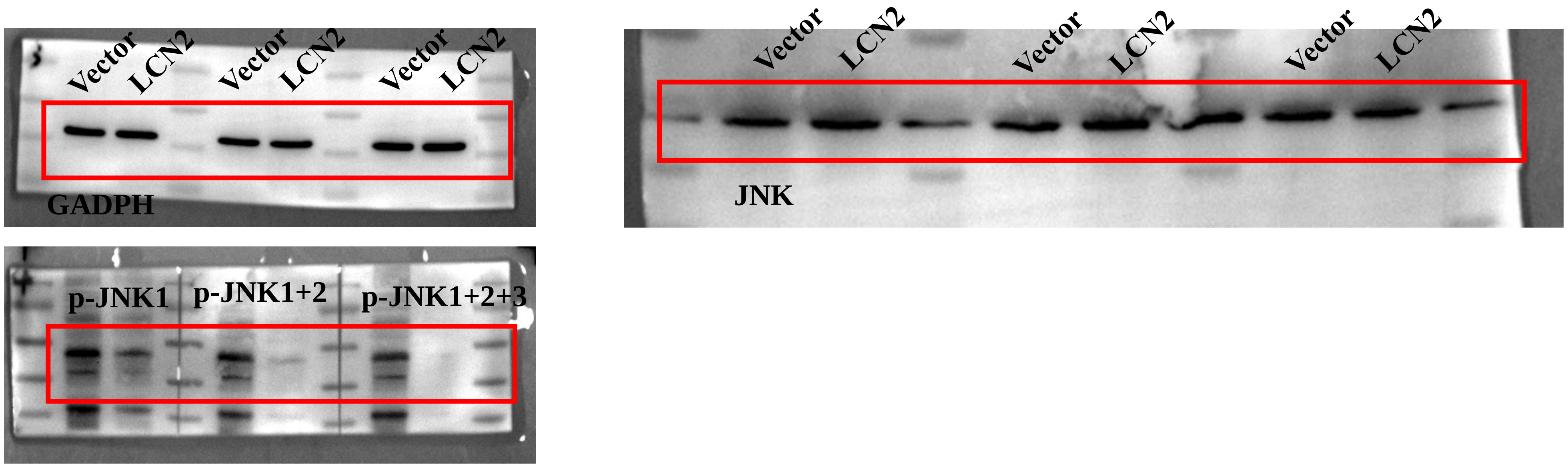

Figure S7

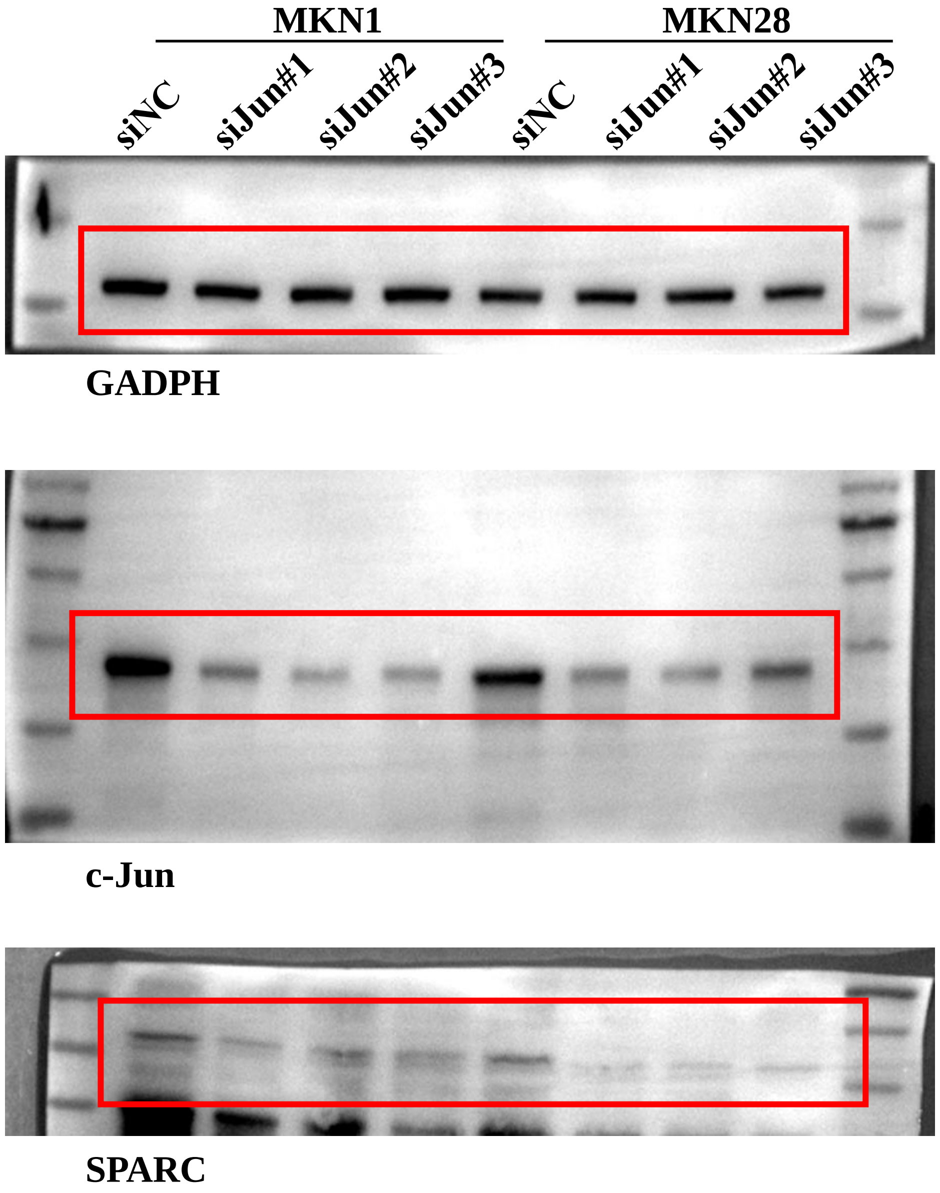

Figure S7C
